# Impact and mechanism of sulphur-deficiency on modern wheat farming nitrogen-related sustainability and gliadin content

**DOI:** 10.1038/s42003-021-02458-7

**Published:** 2021-08-06

**Authors:** Zitong Yu, Maoyun She, Ting Zheng, Dean Diepeveen, Shahidul Islam, Yun Zhao, Yingquan Zhang, Guixiang Tang, Yujuan Zhang, Jingjuan Zhang, Christopher L. Blanchard, Wujun Ma

**Affiliations:** 1 Food Futures Institute, College of Science, Health, Engineering and Education, Murdoch University, Perth, WA Australia; 2 Triticeas Research Institute, Sichuan Agriculture University, Chengdu, China; 3 Department of Primary Industries and Regional Development, South Perth, WA Australia; 4 Institute of Food Science and Technology, Chinese Academy of Agriculture Sciences, Beijing, China; 5 Department of Agronomy, Zhejiang University, Hangzhou, Zhejiang China; 6 ARC Industrial Transformation Training Centre for Functional Grain, Graham Centre for Agricultural Innovation, Charles Sturt University, Wagga Wagga, NSW Australia

**Keywords:** Agriculture, Molecular biology, Plant sciences

## Abstract

Two challenges that the global wheat industry is facing are a lowering nitrogen-use efficiency (NUE) and an increase in the reporting of wheat-protein related health issues. Sulphur deficiencies in soil has also been reported as a global issue. The current study used large-scale field and glasshouse experiments to investigate the sulphur fertilization impacts on sulphur deficient soil. Here we show that sulphur addition increased NUE by more than 20% through regulating glutamine synthetase. Alleviating the soil sulphur deficiency highly significantly reduced the amount of gliadin proteins indicating that soil sulphur levels may be related to the biosynthesis of proteins involved in wheat-induced human pathologies. The sulphur-dependent wheat gluten biosynthesis network was studied using transcriptome analysis and amino acid metabolomic pathway studies. The study concluded that sulphur deficiency in modern farming systems is not only having a profound negative impact on productivity but is also impacting on population health.

## Abstract (web summary)

Zitong Yu et al. utilize large-scale field and glasshouse experiments and transcriptomic and metabolomic analyses to explore the impacts of sulphur fertilization on sulphur-deficient wheat crop and its gluten composition. They show that sulphur fertilization increases nitrogen-use efficiency through regulating glutamine synthetase and reduces the amount of gliadin components through mediating the aspartate-family amino acid biosynthesis network.

## Introduction

Bread wheat (*Triticum aestivum*) end-products are a major food source for humans. Increases in wheat grain yield (GY) and grain protein content (GPC) are required to meet the dietary needs of the growing population. GY is a major determinant of farmer profit, while GPC is an important characteristic of wheat end-products^1^. Simultaneously increasing GY and GPC is challenging due to the negative relationship between the two parameters^2^. To obtain both high GY and GPC, wheat growers tend to use high amounts of nitrogen (N) fertiliser, which reduce N-use efficiency (NUE)^3^. NUE is governed by N uptake from soil, N remobilisation from leaves to grains and N assimilation in developing grains^4^. Finally, N is assimilated into glutamine (Gln) and glutamate (Glu) by glutamine synthetase (GS) and glutamate dehydrogenase through the glutamate synthase/glutamine oxoglutarate aminotransferase cycle (GS/GOGAT)^5–7^.

The suitability of wheat flour for different end-products is determined by its gluten composition. Sulfur is an important nutrient that is involved in determining functional properties of the protein^8^. The relevant metabolic processes start with sulfate uptake by the corresponding transporters, followed by reduction and transfer of the resulting sulfide to activated serine by O-acetylserine lyase to form cysteine (Cys)^9^. Cys is an essential component for the formation of disulfide bonds within the wheat protein matrix, and is therefore important for the development of the viscoelasticity of dough matrix^10,11^. Based on the distribution of cysteine residues, glutens are categorised into three types of subunits: sulfur-poor subunits (ω-gliadins), high molecular weight glutenin subunit (HMW-GS), and sulfur-rich subunits (α/β-gliadins, γ-gliadins, and low molecular weight glutenin subunit (LMW-GS))^12^. The interchain disulfide bonds for the formation of gluten network/gluten macropolymer (GMP) is mainly formed between two cysteine residues of HMW-GS, acting as the chain extender, whereas gliadins have an odd number of cysteine residues, which are linked together or to glutenins, acting as the terminator of glutenins polymerisation^13,14^. Among them, gliadins are the dominant carriers of toxic epitopes, which are responsible for human illness related to wheat gluten consumption including coeliac disease and gluten allergy^15^. Such conditions are induced by oral intake of wheat gluten components rich in proline (Pro), resulting in a reduced susceptibility to protease activity in the gastrointestinal tract. As a result of non-digestion, the peptides rich in Pro and Gln accumulate in the small intestine^16^. Gliadins are divided into three types, including ω-gliadins, α/β-gliadins, γ-gliadins. The glutenins contain 12.33 and 37% of Pro and Gln, respectively, while the gliadins are significantly richer in both, at about 19.75 and 43%, respectively. Notably, ω-gliadins are characterised by the highest content of Gln and Pro, which account for around 80% of the total composition^14,17^. Studies have shown that gliadins are the most toxic component in wheat proteins in relation to coeliac disease, while the glutenins are classified as nontoxic, weakly toxic or not as toxic as gliadins^18–20^. A T-cell stimulatory sequence QQX_1_PX_2_QQ driven by ω5-gliadins has been confirmed as the main toxic epitope, and is the most toxic component inducing wheat-dependent exercise-induced anaphylaxis (WDEIA)^21,22^. For α/β-gliadins, a well-characterised 33-residue peptide from α-gliadins is resistant to gastrointestinal digestion and is used as the marker for coeliac immunoreactivity^23^. The γ-gliadins also contain abundant epitopes^24^. A range of flour treatment approaches have been investigated to reduce the gliadin toxicity, including physical, chemical and enzymatic means^25,26^. Genetic approaches were also used to knock out or knock down the gliadin coding genes. Generally, the gluten extracts from the RNAi line had a between 60 and 80% reduction in total gliadin content and were unable to elicit T-cell responses, but their loaf volume was reduced by between 20 and 30%^27–31^. CRISPR-Cas9 editing on the gene encoding α-gliadins reduced the immunoreactivity by 85%, while it was accompanied by a strong reduction in gluten content of up to 85%^32^. Removing toxic epitopes but maintaining viscoelasticity of wheat flour through genetic modification has been challenging^27–32^.

In the past decades, the soil sulfur content of agricultural land has been depleted, resulting in widespread sulfur deficiency in farming systems in many countries^33,34^. This phenomenon is due to the efficient reduction in sulfur emissions into the atmosphere that result from fossil fuel consumption in most Western countries^35^. The reduction in emissions has resulted in a consequent decrease in atmospheric depositions of sulfur into land used in agricultural production^36^. In addition, modern agricultural practices have resulted in a reduction in soil sulfur inputs and an increase in sulfur outputs. The reduced sulfur inputs are primarily due to the use of highly concentrated fertilisers containing no sulfur, i.e., using triple superphosphate instead of superphosphate. The increased sulfur outputs are the result of intensive cropping systems with high crop yields and a greater removal of sulfur from the soil^37^. As a consequence of the worldwide soil sulfur deficiency, research into plant adaption to sulfur related stresses has been shifted from a focus on acidification by excessive sulfur to the impacts of sulfur deficiency on crop production^38^.

While sulfur has been identified as a key element in regulating plant growth and grain-filling (especially in altering grain protein composition), there are still unanswered questions regarding the impact of soil sulfur deficiency including: (1) Is the soil sulfur deficiency related to the reported lowering NUE? (2) Is the biosynthesis of gluten components affected by sulfur deficiency? (3) How does sulfur regulate the N metabolism and gluten component biosynthesis? The current study addresses these questions and has confirmed that appropriate application of sulfur fertiliser in sulfur deficient farmland has a multi-faceted positive impact on wheat NUE and wheat protein biosynthesis.

## Results

### Sulfur application improves wheat NUE and its underlying mechanism

#### The impacts of sulfur supplementation on agronomically important traits in wheat

**Fig. 1:**
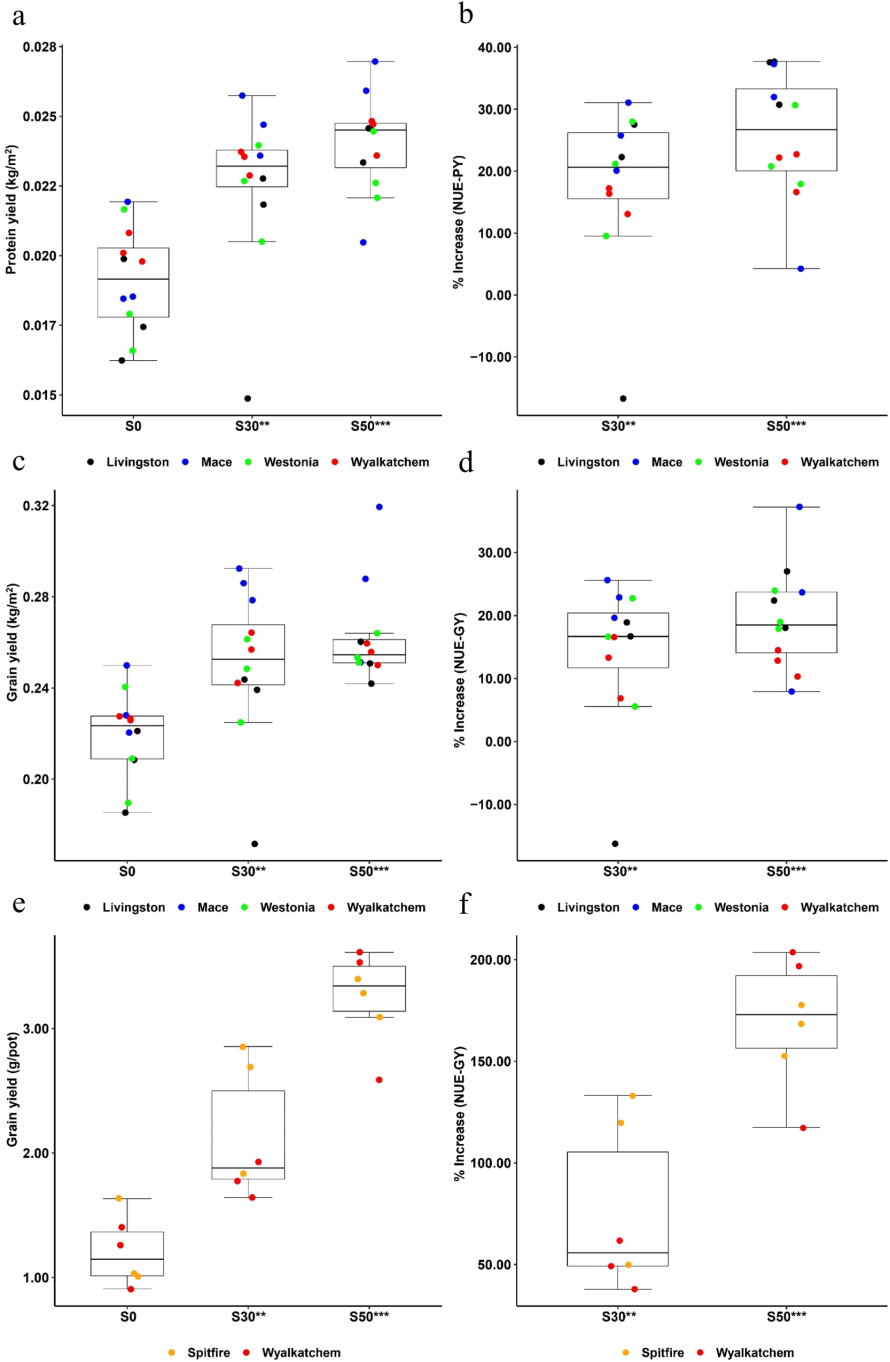
Effects of sulfur treatments on protein yield, grain yield and their corresponding nitrogen-use efficiency improvement in 2014 field trial and 2014 glasshouse experiment. For all panels, S0, S30, S50 mean 0, 30, and 50 kg ha^−1^ sulfur treatments, respectively. *P < 0.05; **P < 0.01; ***P < 0.001; ^ns^not significant; the number of biologically independent replicate (plot or pot) for each cultivar under each sulfur treatment is 3. Boxplots show the median and interquartile ranges (IQR); the end of the top line is the third quartile (Q3) + 1.5 × IQR; the end of the bottom line is the first quartile (Q1) − 1.5 × IQR. The dots with black, blue, green, red and orange respectively show the data distribution of the five cultivars Livingston, Mace, Westonia, Wyalkatchem and Spitfire under each sulfur treatment. The effects of sulfur treatments on PY, GY and their corresponding NUE of each cultivar were shown in Supplementary Fig. 8. **a** The increase in the PY of cultivar Livingston, Mace, Westonia and Wyalkatchem caused by S30 and S50 treatments in 2014 field trial. **b** The percentage of NUE-PY increase for cultivar Livingston, Mace, Westonia and Wyalkatchem at S30 and S50 treatments in 2014 field trial. **c** The increase in the GY of cultivar Livingston, Mace, Westonia and Wyalkatchem caused by S30 and S50 treatments in 2014 field trial. **d** The percentage of NUE-GY increase for cultivar Livingston, Mace, Westonia and Wyalkatchem at S30 and S50 treatments in 2014 field trial. **e** The increase in the GY of cultivar Spitfire and Wyalkatchem caused by S30 and S50 treatments in 2014 glasshouse experiment. **f** The percentage of NUE-GY increase for cultivar Spitfire and Wyalkatchem at S30 and S50 treatments in 2014 glasshouse experiment.

Under a fixed N application rate of 25 kg ha^−1^ in the 2014 field trial, protein yield (PY), GY and their corresponding NUE (NUE-PY and NUE-GY) in the sulfur deficient Western Australia Wheat Belt farming land were significantly affected by supplementation treatments of 30 kg ha^−1^ (S30) and 50 kg ha^−1^ (S50), compared to the no sulfur control (S0) (*P* < 0.01 at S30, *P* < 0.001 at S50, *T*-test, Supplementary Data 1; Table 1, UNIANOVA). Both S30 and S50 treatments resulted in significant increases in grain productivity in comparison with S0 (*P* < 0.01 at S30, *P* < 0.001 at S50, *T*-test, Supplementary Data 1). At S50, the average PY of four cultivars, Livingston, Westonia, Wyalkatchem and Mace, were increased from 0.018 to 0.024 kg m^−2^, 0.019 to 0.023 kg m^−2^, 0.020 to 0.024 kg m^−2^ and 0.020 to 0.024 kg m^−2^, respectively (*P* < 0.001, *T*-test, Supplementary Data 1; Fig. 1a). In comparison with the NUE-PY at S0, the NUE-PY at S50 was significantly increased by 35.4% in Livingston, 23.1% in Westonia, 20.5% in Wyalkatchem and 24.5% in Mace (*P* < 0.001, *T*-test, Supplementary Data 1; Fig. 1b). The average GY of the four cultivars were correspondingly increased from 0.20 to 0.25 kg m^−2^, 0.21 to 0.26 kg m^−2^, 0.23 to 0.26 kg m^−2^ and 0.23 to 0.29 kg m^−2^ (*P* < 0.001, *T*-test, Supplementary Data 1; Fig. 1c). Compared to the NUE-GY at S0, the NUE-GY at S50 was significantly increased by 22.5% in Livingston, 20.3% in Westonia, 12.5% in Wyalkatchem and 22.9% in Mace (*P* < 0.001, *T*-test, Supplementary Data 1; Fig. 1d). Statistically significant impacts of S30 and S50 on GY or NUE-GY in comparison with S0 were also found in the 2014 glasshouse experiments that used soils obtained from the field trial site (*P* < 0.01 at S30, *P* < 0.001 at S50, *T*-test, Supplementary Data 1; Table 1, UNIANOVA). At S50, the average GY of cultivars Spitfire and Wyalkatchem were correspondingly increased from 1.22 to 3.26 g per pot and 1.20 to 3.25 g per pot (*P* < 0.001, *T*-test, Supplementary Data 1; Fig. 1e), indicating significant NUE-GY increases by 166 and 173% in comparison with that at S0, respectively (*P* < 0.001, *T*-test, Supplementary Data 1; Fig. 1f). To explore the morphological aspects that facilitate the NUE-GY improvement, seven peduncle traits related to N remobilisation were measured in 2014 glasshouse experiment^39^. The correlation coefficient values (*r*) showed that, for Spitfire, the GY or NUE-GY was significantly positively correlated with head weight (*r* = 0.9230, *P* < 0.01, Pearson test, Table 2), while for Wyalkathcem, they are significantly correlated with straw diameter (*r* = 0.7658, *P* < 0.05, Pearson test, Table 2), keycard diameter (*r* = 0.6706, *P* < 0.05, Pearson test, Table 2) and distance between neck and card (*r* = 0.7882, *P* < 0.05, Pearson test). Under sulfur treatment, the four peduncle traits were all significantly enhanced (*P* < 0.05, *T*-test, Supplementary Data 1; Table 1, UNIANOVA; Supplementary Fig. 1a–d), indicating that the increases in NUE-GY through sulfur application are associated with the enhancement of these peduncle traits. In view of both field trial and glasshouse experiment results, sulfur application can significantly increase PY (NUE-PY) and GY (NUE-GY) (*P* < 0.01 at S30, *P* < 0.001 at S50, *T*-test, Supplementary Data 1; Table 1, UNIANOVA).

**Table 1:** Univariate analysis of variance of the impact of the genotypes (G), sulfur treatments (E) and G×E on agronomically important traits in 2014 field trial and glasshouse experiment.

		Protein yield	Grain yield	Peduncle length	Neck to card	Straw diameter	Keycard diameter	Head weight
Field trial	G*E	0.75^ns^	0.78^ns^					
	Genotype	2.45^ns^	7.64**					
	Treatment	17.45***	15.07***					
Glasshouse experiment	G*E		1.50^ns^	2.17^ns^	5.09*	2.62^ns^	2.36^ns^	2.14^ns^
	Genotype		1.84^ns^	9.68**	1.26^ns^	3.91^ns^	1.72^ns^	11.43**
	Treatment		43.81***	15.15**	7.08**	16.59***	24.19***	50.90***

The SPSS univariate analysis of variance (UNIANOVA) in general linear model was used to calculate the *F*-values of the four genotypes (G), three sulfur treatments (E) and G×E on each agronomically important trait. *P < 0.05, **P < 0.01, ***P < 0.001; ^ns^means not significant; the number of biologically independent replicate (plot or pot) for each genotype under each sulfur treatment is 3. Under a fixed nitrogen application rate of 25 kg ha^-1^, the statistically significant impacts of sulfur treatments on protein yield, grain yield (nitrogen-use efficiency) and morphologic traits were found in both 2014 field trial and glasshouse experiment.

**Table 2:** Correlation coefficient values *r* of peduncle traits and grain yield (nitrogen-use efficiency) for Spitfire and Wyalkatchem under three sulfur treatments in 2014 glasshouse experiment.

	Neck diameter	Head weight	Peduncle length	Straw diameter	Keycard diameter	Neck to card	Straw weight
Spitfire	−0.5522^ns^	0.9230**	0.6649^ns^	0.4710^ns^	0.4674^ns^	0.6052^ns^	0.4064^ns^
Wyalkatchem	0.4959^ns^	0.5812^ns^	0.5771^ns^	0.7658*	0.6706*	0.7882*	0.2861^ns^

The sample size of each cultivar is 9, including samples collected from three sulfur treatments with three biologically independent replicates (or three pots) for each sulfur treatment; the number of biologically independent replicate (or pot) for each cultivar under each sulfur treatment is 3; *r* > 0.666, *P* < 0.05; *r* > 0.798, *P* < 0.01; *P < 0.05, **P < 0.01; ^ns^not significant. The correlation coefficient values *r* of head weight for Spitfire, straw diameter, keycard diameter, and neck to card distance for Wyalkatchem with grain yield (nitrogen-use efficiency) were statistically significant in 2014 glasshouse experiment.

#### The regulatory mechanism behind sulfur-mediated improvement in wheat NUE

The field trial and glasshouse experiment demonstrated that an increase in NUE can be achieved by sulfur fertilisation. To understand how N metabolism is affected by sulfur availability, a widely adopted bread wheat cultivar, Spitfire, was selected for further study using comparative transcriptome analysis and free amino acid dynamics across different grain development stages. Enzyme activity dynamics across different plant growth and seed development stages was also studied. Similar results on NUE improvement under S30 treatment were obtained between the 2014 and 2015 glasshouse experiments^40^. A preliminary comparative transcriptome analysis using developing grain samples of 7 days post-anthesis (DPA) revealed that three differentially expressed genes (DEGs) downregulated by S30, and annotated as glutamine synthetase (GS) were enriched in N metabolism-related GO items and KEGG pathways^40^. The expression patterns of these three DEGs in S0 and S30 treatments across three grain developing stages were analysed in the current study, which were nearly identical with *glutamine synthetase 1* (*GS1*), but not with *GS2* (Supplementary Fig. 2 and Supplementary Data 2), indicating that these three DEGs were specifically *GS1* and that the pivotal enzyme GS in the N assimilation pathway is regulated by sulfur availability. These results indicate that GS is the major connection between sulfur and N metabolism.

**Fig. 2:**
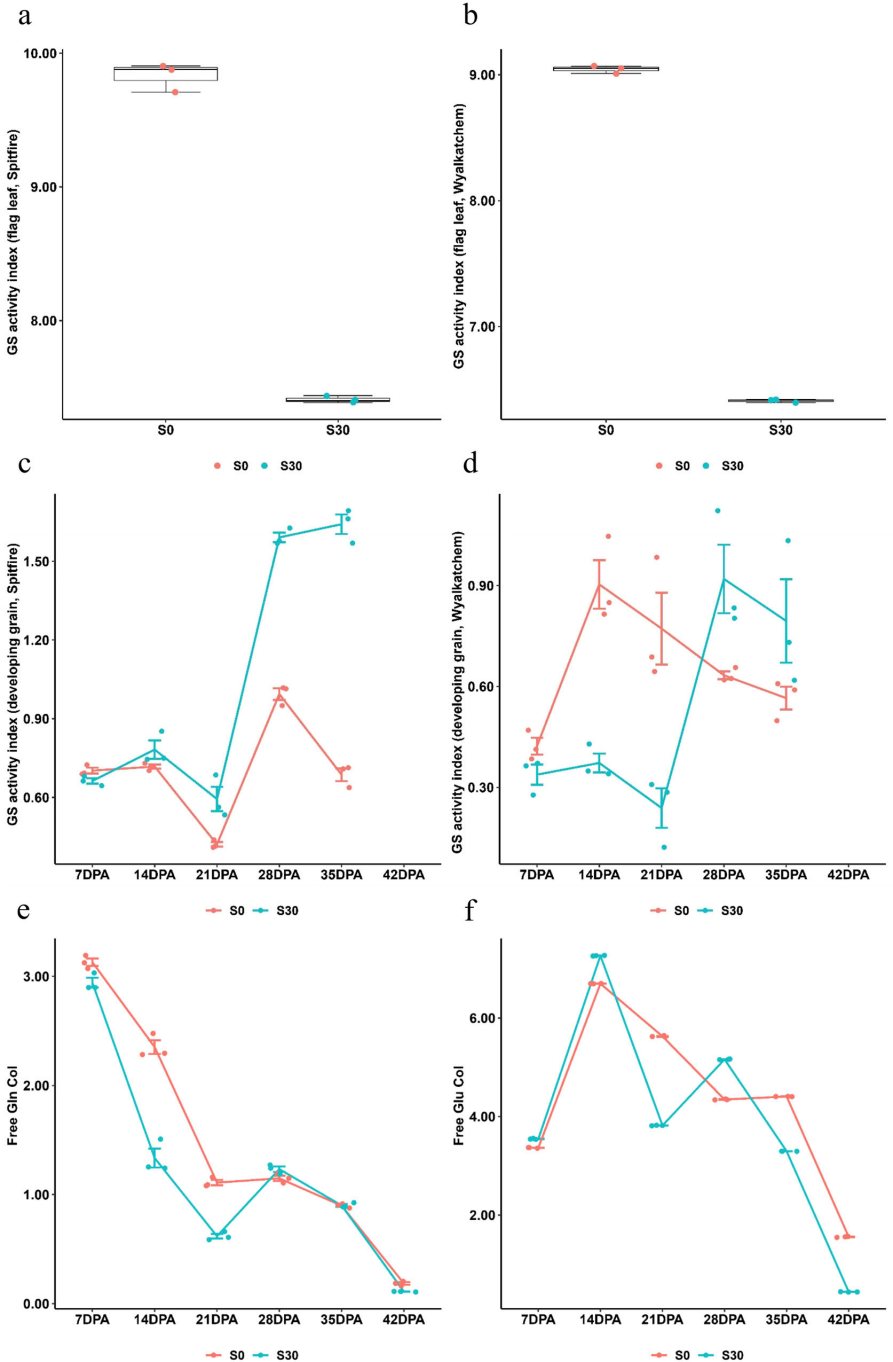
Comparison of glutamine synthetase activity, free glutamine and free glutamic acid contents in flag leaves and developing grains between different sulfur treatments in 2015 glasshouse experiment. For all panels, S0, S30 mean 0 and 30 kg ha^−1^ sulfur treatments, respectively. GS glutamine synthetase, GS activity index: GS activity × 10^5^, DPA days post-anthesis, CoI content index (free amino acid% × 10^4^). **a**, **b** The flag leaf samples from three replicate pots under the same sulfur treatment were pooled together, making it 2 pooled flag leaf samples with three technical repeats each. **c**–**f** The developing grain samples of three replicate pots from the same grain developing stage and sulfur treatment were pooled together, making it 10 pooled developing grain samples with three technical repeats each. Boxplots in **a**, **b** show the median and interquartile ranges (IQR); the end of the top line is the third quartile (Q3) + 1.5 × IQR; the end of the bottom line is the first quartile (Q1) − 1.5 × IQR; the dots with red and green respectively show the data distribution under S0 and S30 treatment; Line graphs in **c**–**f** show the means ± standard deviation of three technical repeats with a mix of three biologically independent replicates (or three pots); the dots with red and green show the data distribution under S0 and S30, respectively. **a** The comparison of GS activity in flag leaves of Spitfire between S0 (red) and S30 (green). **b** The comparison of GS activity in flag leaves of Wyalkatchem between S0 (red) and S30 (green). **c** The comparison of GS activity in developing grains of Spitfire between S0 (red) and S30 (green). **d** The comparison of GS activity in developing grains of Wyalkatchem between S0 (red) and S30 (green). **e** The comparison of free glutamine (Gln) content in developing grains of Spitfire between S0 (red) and S30 (green). **f** The comparison of free glutamic acid (Glu) content in developing grains of Spitfire between S0 (red) and S30 (green).

The dynamics of GS activity in flag leaves and developing grains of Spitfire and Wyalkatchem from the 2015 glasshouse experiment were compared between S0 and S30 treatment. In flag leaves, GS activity of both cultivars was greater at S0 than S30 (Fig. 2a, b). In developing grains from 7 to 21 DPA, the GS activity was lower at S30 than S0 for Wyalkatchem but was slightly higher for Spitfire. As grain continued to develop, the GS activity in both cultivars gradually increased with sulfur supplementation. From 28 to 35 DPA, the GS activity of both cultivars was much higher at S30 than S0 (Fig. 2c, d). These results suggest that the utilisation of free Gln during grain development was increased with sulfur supplementation, which is reflected by the differences in the dynamics of free Gln and Glu content between S0 and S30 during grain-filling (Fig. 2e, f).

### Sulfur application reduces gliadin content

#### Changes in gliadin content with soil sulfur supplementation

**Fig. 3:**
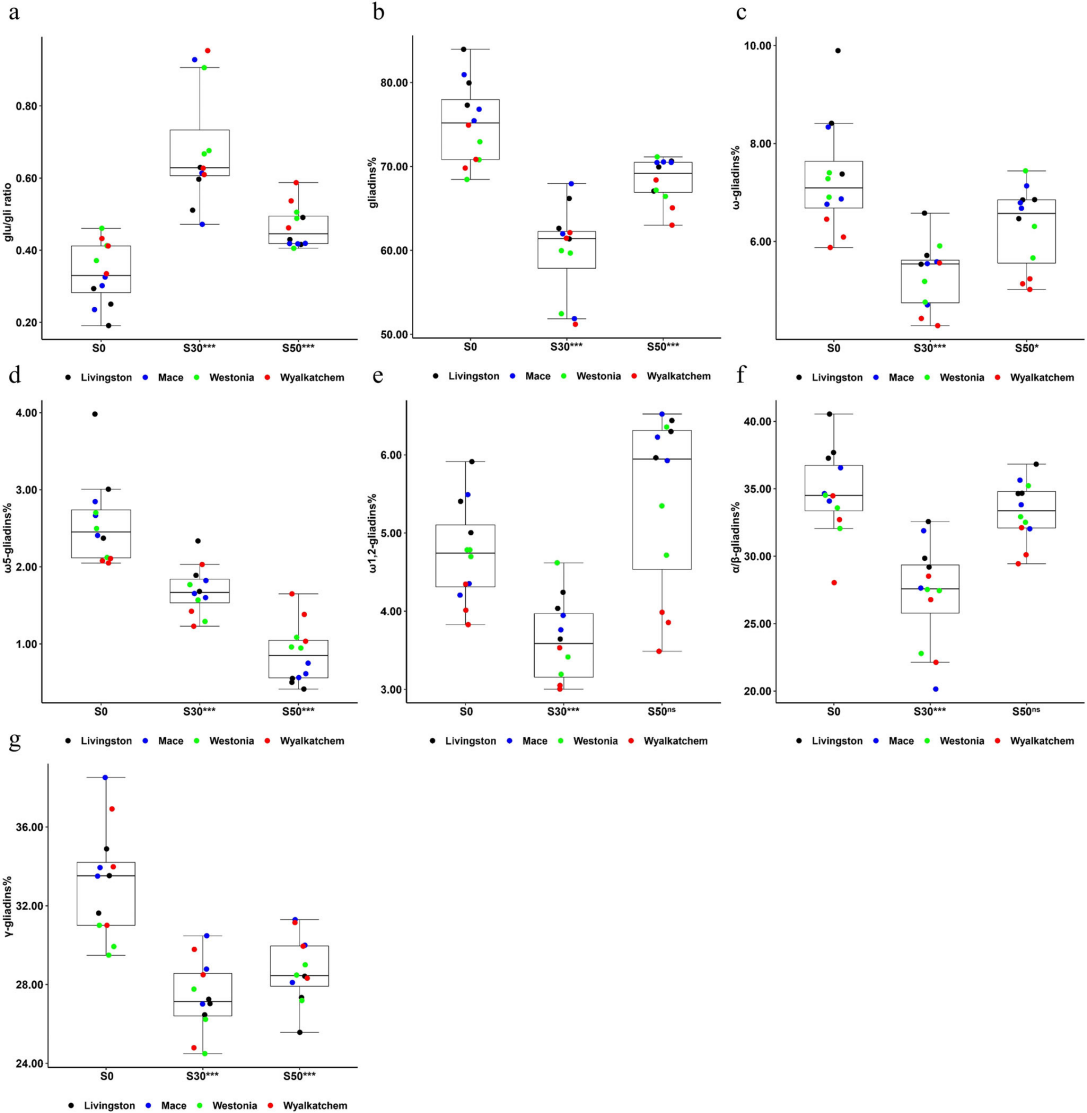
Effects of sulfur treatments on the ratio of glutenin to gliadin, the percentages of gliadins and its each subtype in the total gluten content in 2014 field trial. For all panels, S0, S30, S50 mean 0, 30 and 50 kg ha^−1^ sulfur treatments, respectively; *P < 0.05; **P < 0.01; ***P < 0.001; ^ns^not significant; the number of biologically independent replicate (or plot) for each cultivar under each sulfur treatment is 3. Boxplot shows the median and interquartile ranges (IQR); the end of the top line is the third quartile (Q3) + 1.5 × IQR; the end of the bottom line is the first quartile (Q1) − 1.5 × IQR. The dots with black, blue, green and red show the data distribution of the four cultivars Livingston, Mace, Westonia and Wyalkatchem under each sulfur treatment. The effects of sulfur treatments on the ratio of glutenin to gliadin, the percentages of gliadins and its each subtype in the total gluten content of each cultivar were shown in Supplementary Fig. 9. **a** The changes in glu/gli ratio of the four cultivars caused by S30 and S50 treatments. **b** The changes in the percentage of gliadins in the total gluten content (gliadins%) of the four cultivars caused by S30 and S50 treatments. **c** The changes in the percentage of ω-gliadins in the total gluten content (ω-gliadins%) of the four cultivars caused by S30 and S50 treatments. **d** The changes in the percentage of ω5-gliadins in the total gluten content (ω5-gliadins%) of the four cultivars caused by S30 and S50 treatments. **e** The changes in the percentage of ω1,2-gliadins in the total gluten content (ω1,2-gliadins%) of the four cultivars caused by S30 and S50 treatments. **f** The changes in the percentage of α/β-gliadins in the total gluten content (α/β-gliadins%) of the four cultivars caused by S30 and S50 treatments. **g** The changes in the percentage of γ-gliadins in the total gluten content (γ-gliadins%) of the four cultivars caused by S30 and S50 treatments.

Under a fixed N application rate of 25 kg ha^−1^ in the 2014 field trial, statistically significant impacts of sulfur fertilisation on the ratio of glutenin to gliadin (glu/gli ratio) and the percentages of gliadins and its subtypes (ω-, α/β-, γ-) in the total gluten content of the four wheat cultivars were observed (*P* < 0.05, *T*-test, Supplementary Data 1 and Table 3, UNIANOVA). No significant changes were observed for the total gluten content. The average glu/gli ratio of cultivars Livingston, Mace, Westonia and Wyalkatchem were all increased by sulfur application at S30 from 0.25 to 0.58, 0.29 to 0.67, 0.41 to 0.75 and 0.39 to 0.73, respectively, but reverted to 0.45, 0.42, 0.47 and 0.54, respectively, when the sulfur application was increased to 50 kg ha^−1^ (S50) (*P* < 0.001 at S30 and S50, *T*-test, Supplementary Data 1 and Fig. 3a). Consequently, the appropriate sulfur application S30 decreased the average percentage of gliadins in the total gluten of the four cultivars from 80.4 to 63.4%, 77.7 to 60.6%, 70.7 to 57.4% and 71.9 to 58.3%, respectively, which represented decreases of 21.1%, 22.0%, 18.8% and 18.9%, respectively (*P* < 0.001 at S30, *T*-test, Supplementary Data 1 and Fig. 3b). The same trends were observed for the percentage of each gliadin subtype in the total gluten content in relation to the three sulfur regimes. The average ω-gliadins% of the four cultivars was reduced by S30 from 8.6 to 5.9%, 7.3 to 5.3%, 7.2 to 5.3% and 6.1 to 4.8%, which represented decreases of 31.4%, 27.4%, 26.4% and 21.3%, respectively; while the magnitude of the reduction was lower at S50, by 21.2%, 5.5%, 9.7% and 16.4%, respectively (*P* < 0.001 at S30, *P* < 0.05 at S50, *T*-test, Supplementary Data 1 and Fig. 3c). The average ω5-gliadin% of the four cultivars was reduced from 3.1 to 2.0%, 2.6 to 1.7%, 2.4 to 1.5% and 2.1 to 1.6% by S30, respectively, which represented a decrease of 35.5, 34.6, 37.5 and 23.8%. At S50, the magnitude of the reductions was even greater at 83.9%, 76.9%, 58.3% and 33.3%, respectively (*P* < 0.001 at S30 and S50, *T*-test, Supplementary Data 1 and Fig. 3d). The average ω1,2-gliadins% of the four cultivars was reduced at S30 from 5.4 to 4.0%, 4.7 to 3.6%, 4.8 to 3.7% and 4.1 to 3.2%, respectively, which represented decreases of 25.9, 23.4, 22.9 and 22.0%. At S50, the average ω1,2-gliadins% was increased by 14.8%, 31.9%, 14.6% and −7.3%, respectively (*P* < 0.001 at S30, *P* > 0.05 at S50, *T*-test, Supplementary Data 1 and Fig. 3e). The average α/β-gliadins% of cultivars Livingston, Mace, Westonia and Wyalkatchem were reduced from 38.5 to 30.5%, 35.1 to 26.6%, 33.4 to 25.9% and 31.7 to 25.8% by S30, respectively, which represented decreases of 20.8, 24.2, 22.5 and 18.6%. At S50, three cultivars, Livingston, Mace, Wyalkatchem, saw reductions of 8.1, 3.7 and 3.5%, while for Westonia there was an increase of 0.6% (*P* < 0.001 at S30, *P* > 0.05 at S50, *T*-test, Supplementary Data 1 and Fig. 3f). The average γ-gliadins% of the four cultivars was reduced from 33.3 to 26.9%, 35.3 to 28.8%, 30.1 to 26.2% and 34.0 to 27.7% at S30, respectively, which represented decreases of 19.2, 18.4, 13.0 and 18.5%. At S50, the decrease were 18.6, 15.6, 6.3 and 12.4%, which were slightly less than at S30 (*P* < 0.001 at S30 and S50, *T*-test, Supplementary Data 1 and Fig. 3g).

**Table 3:** Univariate analysis of variance of the impact of the genotypes (G), sulfur treatments (E) and G×E on gluten components in 2014 field trial and glasshouse experiment.

		Glu/gli ratio	Gliadins%	ω5-gliadins%	ω1,2-gliadins%	ω-gliadins%	α/β-gliadins%	γ-gliadins%
Field trial	G*E	0.39^ns^	0.77^ns^	4.93**	3.22*	1.10^ns^	0.39^ns^	0.90^ns^
	Genotype	3.25*	5.25**	0.84^ns^	17.38***	11.53***	6.77**	5.23**
	Treatment	33.63***	47.66***	81.96***	44.69***	29.39***	27.48***	33.44***
Glasshouse experiment	G*E	6.88*	7.47**	2.52^ns^	1.42^ns^	3.87*	2.61^ns^	6.64*
	Genotype	7.98*	9.93**	0.07^ns^	1.77^ns^	0.85^ns^	0.43^ns^	45.45***
	Treatment	26.25***	37.13***	23.86***	17.26***	26.75***	24.38***	18.79***

The SPSS univariate analysis of variance (UNIANOVA) in general linear model was used to calculate the *F*-values of the four genotypes (G), three sulfur treatments (E) and G×E on each gluten component. *P < 0.05, **P < 0.01, ***P < 0.001; ^ns^not significant; the number of biologically independent replicate (plot or pot) for each genotype under each sulfur treatment is 3. Under a fixed nitrogen application rate of 25 kg ha^−1^, the statistically significant impacts of sulfur treatments on the ratio of glutenin to gliadin (glu/gli ratio), the percentages of gliadins (gliadins%) and its each subtype (ω-gliadins%, α/β-gliadins% and γ-gliadins%) in the total gluten content were observed in both 2014 field trial and glasshouse experiment.

In summary, with 25 kg ha^−1^ N application in 2014 field trial, an appropriate level of sulfur application was shown to reduce gliadin biosynthesis and accumulation. Among the gliadin fractions, the highest reduction of ω-gliadins was found at S30. However, for the most toxic gliadin component for WDEIA, the ω5-gliadins, its highest reduction was found at S50, with a reduction as high as 83.9%.

#### The regulatory mechanism behind sulfur-mediated gluten component biosynthesis

*Gene interaction network for ABA responses to sulfur availability during grain-filling:* To understand how gluten component biosynthesis is affected by sulfur availability, the comparative transcriptomic analysis was conducted among three grain-filling stages (7, 14 and 21 DPA), including 14 vs. 7 DPA and 21 vs. 14 DPA for both S0 (LS) and S30 (HS) using the developing grains from 2015 glasshouse experiment. A total of 20,997 grain developmental DEGs were identified in four libraries, including 5012 DEGs in library HS14 vs. HS7, 2676 DEGs in library HS21 vs. HS14, 9501 DEGs in library LS14 vs. LS7, and 3808 DEGs in library LS21 vs. LS14 (Supplementary Fig. 3). Among them, a total of 1004 DEGs were identified from the significantly enriched GO items and KEGG pathways (corrected *P*-value ≤ 0.05), including 365 DEGs in library HS14 vs. HS7, 492 DEGs in library HS21 vs. HS14, 502 DEGs in library LS14 vs. LS7, and 595 DEGs in library LS21 vs. LS14 (Supplementary Data 3). The 1000 bp promoter sequence before the transcription start site (ATG) of the 1004 DEGs were collected from BioMart in Ensembl Plants as their core promoter regions (http://plants.ensembl.org/biomart/martview) (Supplementary Data 4). All 1004 promoter sequences were searched against a *cis*-acting element database from PlantPAN 2.0 with a threshold of support at 100% (Supplementary Data 5). Based on this analysis, a total of 55 *cis*-acting elements were identified. However, considering the 0.02% error rate during sequencing, motifs appearing at a percentage less than 0.02% in the 1004-promoter sequence pool were removed. Finally, 40 valid *cis*-acting elements were identified for six phytohormone responders in response to sulfur availability during three grain-filling stages (Supplementary Data 6). A total of 18 out of the 40 *cis*-acting elements acted as ABA responders. Among the 1004 DEGs, 416 DEGs were differentially expressed in four libraries that were specifically constructed based on two comparisons of grain development stages (14 vs. 7 DPA and 21 vs. 14 DPA) for two sulfur treatment levels (S30 and S0), including 412 DEGs in library HS14 vs. HS7, 411 DEGs in library HS21 vs. HS14, 414 DEGs in library LS14 vs. LS7 and 410 DEGs in library LS21 vs. LS14 (Supplementary Data 7). The promoter regions of the 416 DEGs contained 18 ABA responsive *cis*-acting elements. Meanwhile, DEGs encoding the corresponding transcription factors (264) were filtered out based on the differentially expressed patterns in these four libraries (Supplementary Data 8). Therefore, a total of 680 DEGs were integrated for gene interaction network construction using the ‘Gene prioritisation based on network direct neighbourhood’ function within WheatNet^41^. The resulting interaction network showed a total of 225 connected gene groups. Among them, 52 genes encoding four transcription factor families interacted directly with another 50 genes (Supplementary Fig. 4). Hierarchical clustering classified these 102 genes into two primary groups, based on their expressed patterns across 7, 14 and 21 DPA in S0 and S30. Each group was subdivided into two sub-clusters (Supplementary Fig. 5).

**Fig. 4:**
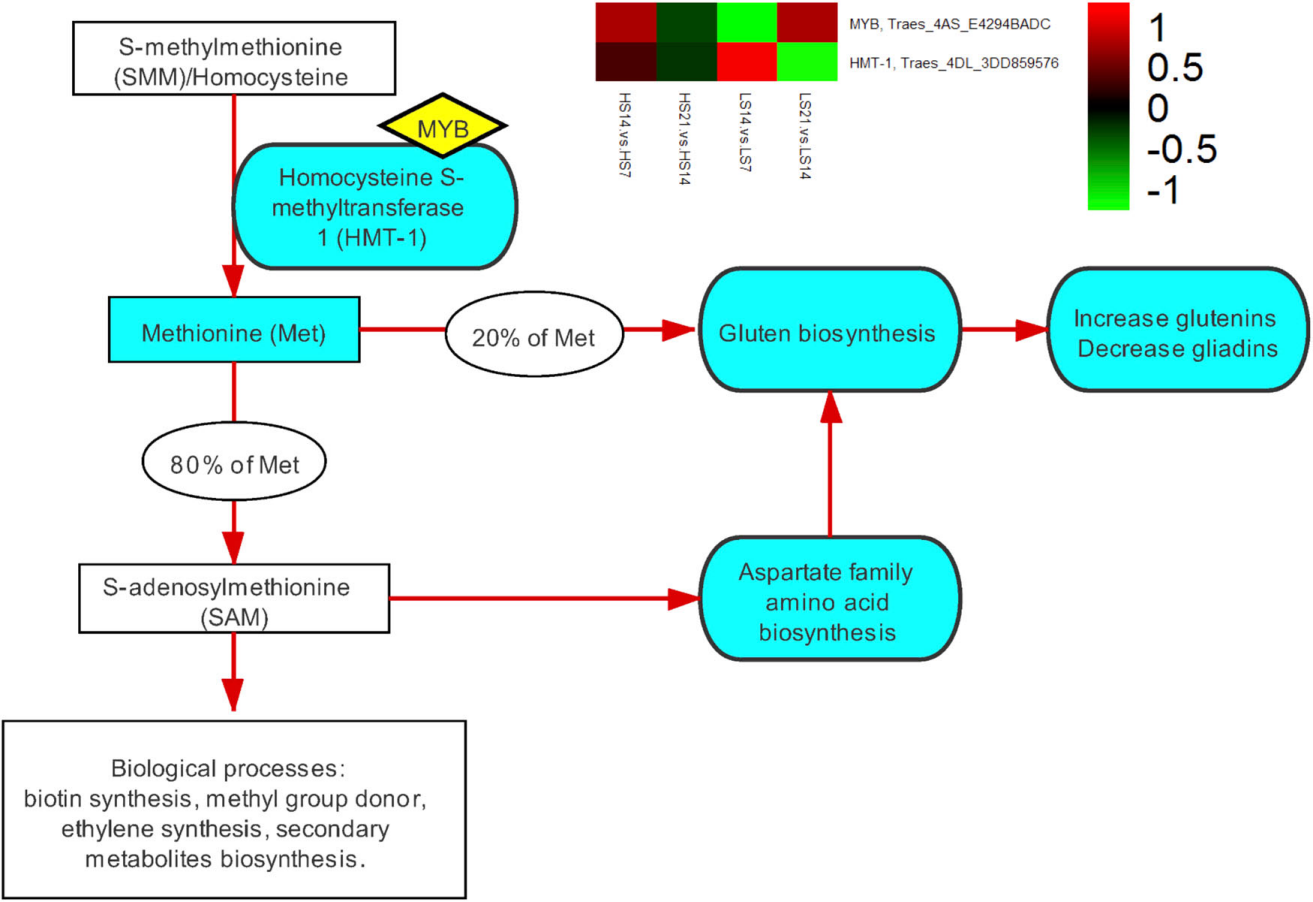
Sulfur-mediated molecular mechanism for methionine biosynthesis. LS (S0) and HS (S30) mean 0 and 30 kg ha^−1^ sulfur treatments, respectively; Met methionine, HMT-1 homocysteine S-methyltransferase 1, DPA days post-anthesis, HS7: S30 at 7 DPA, HS14: S30 at 14 DPA, HS21: S30 at 21 DPA, LS7: S0 at 7 DPA, LS14: S0 at 14 DPA, LS21: S0 at 21 DPA; the heatmap: each row represents a single gene and each column represents a library constructed between two grain developing stages at S0 or S30; the heatmap colour scale: red, green and black indicate upregulation, downregulation and no change, respectively. The gene annotated as MYB (*TraesCS4A02G124900*) was directly interacting with the gene *TraesCS4D02G242100* annotated as HMT-1 in the regulation of Met biosynthesis as a part of the aspartate-family amino acid biosynthesis network. At S30, the expression of the two genes is simultaneously upregulated as grain developing until 14 DPA, followed by a concurrently downregulated until 21 DPA; while at S0, the expression of MYB is downregulated but the expression of HMT-1 is upregulated from 7 to 14 DPA, followed by a reversed pattern from 14 to 21 DPA.

*Sulfur-mediated molecular mechanism for methionine (Met) biosynthesis*: Based on the gene interaction network (Supplementary Fig. 4) and the hierarchical clustering of the 102 DEGs (Supplementary Fig. 5), the gene annotated as MYB (*TraesCS4A02G124900*) was directly interacting with the gene *TraesCS4D02G242100* annotated as homocysteine S-methyltransferase 1 (HMT-1) and located on chromosome 4D in the regulation of Met biosynthesis as part of the aspartate-family amino acid biosynthesis network (Fig. 4). At S30, the expression of the two genes is simultaneously upregulated during grain development until 14 DPA, followed by a subsequent downregulation until 21 DPA. At S0, the expression of MYB is downregulated but the expression of HMT-1 is upregulated from 7 to 14 DPA, followed by a reversed pattern from 14 to 21 DPA (Fig. 4). These expression patterns indicate that they may work synergistically as an activator at S30 and as a repressor at S0.

**Fig. 5:**
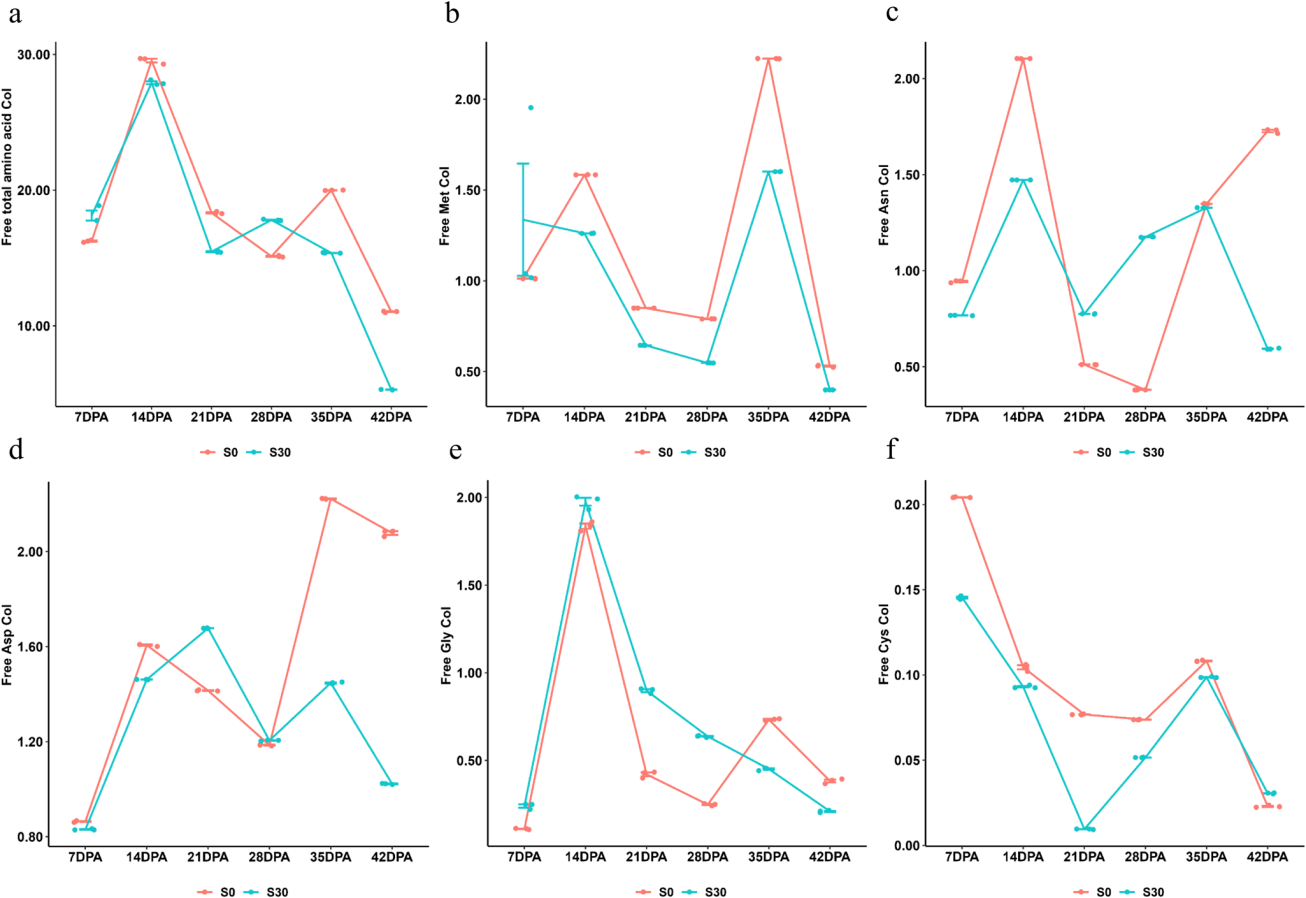
Comparison of six free amino acid dynamics between different sulfur treatments across six grain-filling stages in 2015 glasshouse experiment. Detailed information is shown in the Supplementary Note 1. For all panels, the S0 (red) and S30 (green) mean 0 and 30 kg ha^−1^ sulfur treatments, respectively; DPA days post-anthesis, CoI content index (free amino acid% × 10^4^). The developing grain samples of three replicate pots from the same grain developing stage and sulfur treatment were pooled together, making it 12 pooled developing grain samples with three technical repeats each; line graph shows the means ± 3 technical repeats with a mix of three biologically independent replicates (or three pots); the dots with red and green show the data distribution of Spitfire under S0 and S30, respectively. **a** The comparison of the dynamics of free total amino acid CoI between S0 and S30 treatments during grain-filling. **b** The comparison of the dynamics of free methionine (Met) CoI between S0 and S30 treatments during grain-filling. **c** The comparison of the dynamics of free asparagine (Asn) CoI between S0 and S30 treatments during grain-filling. **d** The comparison of the dynamics of free aspartic acid (Asp) CoI between S0 and S30 treatments during grain-filling. **e** The comparison of the dynamics of free glycine (Gly) CoI between S0 and S30 treatments during grain-filling. **f** The comparison of the dynamics of free cysteine (Cys) CoI between S0 and S30 treatments during grain-filling.

*Free amino acid content dynamics in response to sulfur availability during grain-filling*: The detailed information for the dynamics of six free amino acids is shown in the Supplementary Note 1. The free total amino acid Content Index (CoI, free total amino acid% × 10^4^) was slightly higher in S30 than in S0 at 7 DPA, followed by a similar pattern during 14 to 21 DPA and a sharper decrease in S30 than in S0 from 21 to 42 DPA, resulting in less free amino acid residuals remaining in the mature grain under S30 treatment. These dynamic patterns indicate that sulfur application impacted the free amino acid levels in developing grains (Fig. 5a). Four amino acids were found to be involved in the proposed sulfur-regulated gluten component biosynthesis network, including Met, asparagine (Asn), aspartic acid (Asp) and glycine (Gly). For the sulfur containing amino acid Met, its free CoI (free Met% × 10^4^) in S30, which was much higher than in S0 at 7 DPA, followed by a decrease in S30 and an increase in S0 during 7 to 14 DPA. Even though the two treatments showed a similar pattern from 14 to 42 DPA, the Met residues in mature grains (42 DPA) in S30 was less than in S0, indicating that sulfur application promotes more free Met to be synthesised into the downstream metabolites rather than accumulating in the cell (Fig. 5b). The dynamics of other aspartate-family amino acids including Asn, Asp and Gly were also compared between S30 and S0 treatments. The free Asn CoI (free Asn% × 10^4^) in S30 was slightly lower than in S0 at 7 DPA, and was nearly the same between the two treatments at 35 DPA. A large difference between the two treatments was observed from 35 to 42 DPA, with an obvious decrease at S30 and a reversed increase at S0 (Fig. 5c). For free Asp CoI (free Asp% × 10^4^), no significant difference was observed between the two treatments during 7 to 28 DPA, while a slight decrease occurred at S30 but an increase was found to occur at S0 from 28 to 42 DPA (Fig. 5d). Both Asn and Asp residues in the mature grains of S30 present in lower levels than in S0, indicating that under sulfur application, the free Asn and Asp are more available to participate in the aspartate-family amino acid biosynthesis. For the essential amino acid for gluten component biosynthesis, Gly, there was no obvious difference in its free CoI (free Gly% × 10^4^) between the two treatments during 7 to 14 DPA. While a small fluctuate occurred to S0 from 14 to 42 DPA, the free Gly CoI was continued to decrease at S30, indicating that the sulfur application promotes free Gly to take part in gluten biosynthesis during grain-filling (Fig. 5e). To examine the impacts of sulfur availability on the formation of intra/inter disulfide bonds between two cysteine residues for gluten polymerisation, the dynamics of free Cys content was compared between S30 and S0. At the beginning of grain-filling from 7 to 21 DPA, the free Cys CoI (free Cys% × 10^4^) in S30 was lower than that in S0, followed by a larger decrease in S30 than in S0. During the 21 to 35 DPA period, the free Cys CoI increased slightly in S0 and sharply in S30 (Fig. 5f). The dynamic changes of free Cys contents in S30 appeared to be more variable than in S0 during grain-filling.

**Fig. 6:**
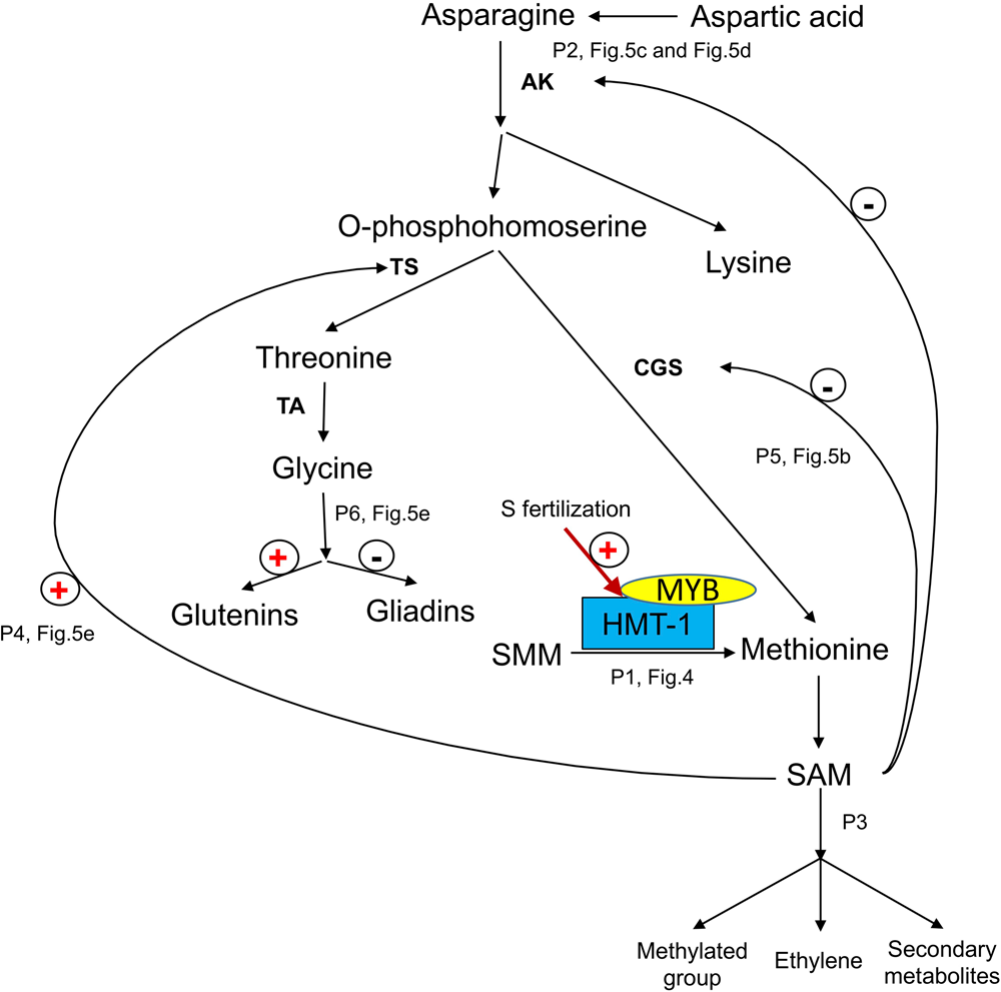
Sulfur-mediated regulatory network for gluten component biosynthesis. Asn asparagine, Asp aspartic acid, AK aspartic acid kinase, CGS cystathionine γ-synthase, Gly glycine, Met methionine, SMM S-methylmethionine, SAM S-adenosylmethionine, TS threonine synthase, TA threonine aldolase, Thr threonine, HMT-1 homocysteine S-methyltransferase 1, DPA days post-anthesis. P1: sulfur promotes MYB acting as a synergistic activator when interacting with HMT-1 to mediate the conversion of SMM to Met and then to SAM (Fig. 4); P2: at the beginning of grain-filling, the amount of free Asn and Asp in sulfur-treated grain is the same as that in the control, but then become more dynamic under sulfur treatment, and their residues in sulfur-treated mature grain are much lower than those of the control, which show that the SAM promoted by sulfur does not accumulate in the cell to inhibit the activity of AK in catalysing aspartate into other amino acids (Fig. 5c, d); P3: the SAM undergoes conversion into secondary metabolites to participate in grain development; P4: SAM stimulates the activity of TS to produce Thr, followed by the biosynthesis of Gly through the catalyzation of TA (Fig. 5e); P5: SAM negatively regulates the major regulatory enzyme of Met biosynthesis CGS (Fig. 5b); P6: TS and CGS compete for the common substrate O-phosphohomoserine. Thus, under sulfur treatment, metabolic flux is prone to Thr synthesis for the subsequent Gly synthesis rather than Met synthesis, as reflected by the differences in the free Met content between the two treatments during grain-filling (Fig. 5b). The free Gly content in S30 is higher than in S0 at the beginning of grain-filling from 7 to 14 DPA, while it continues to decrease, making its residue in mature grain under S30 less than that of S0 (Fig. 5e). The different content of Gly between glutenins and gliadins marks the major difference between the two gluten subtypes, being 13.33% in glutenins and 1.75% in gliadins. Thus, a higher free Gly content caused by sulfur treatment is more advantageous to glutenin biosynthesis but is less favourable for gliadin biosynthesis (Fig. 5e).

*Sulfur-mediated regulatory network for gluten component biosynthesis*: The above experimental results revealed the sulfur-mediated regulatory network for gluten component biosynthesis (Fig. 6). In this network, sulfur promotes MYB acting as a synergistic activator when interacting with HMT-1 to mediate the conversion of S-methylmethionine (SMM) to Met and then S-adenosylmethionine (SAM) (Figs. 4 and 6-P1). At the beginning of grain-filling, the amount of free Asn and Asp in sulfur-treated grain is the same as that of the control, but then became more dynamic under sulfur treatment, and their residues in sulfur-treated mature grain were substantially less than those of the control (Figs. 5c, d and 6-P2). This showed that the SAM promoted by sulfur is inclined to undergo conversion into secondary metabolites to participate in grain developing, rather than accumulating in the cell to inhibit the activity of aspartic acid kinase (AK) to catalyse aspartate into other amino acids. This indicates that high sulfur availability is advantageous to engage SAM in the biological processes of grain development as the precursor of numerous plant essential metabolites and methyl group donors^42^ (Fig. 6-P3). SAM stimulates the activity of threonine synthase (TS) to produce threonine (Thr), followed by the biosynthesis of Gly through the catalyzation of threonine aldolase (TA)^43^ (Figs. 5e and 6-P4). SAM also negatively regulates the major regulatory enzyme of the Met biosynthesis, cystathionine γ-synthase (CGS)^44^ (Figs. 5b and 6-P5). TS and CGS compete for the common substrate O-phosphohomoserine, the precursor of Thr and Met^45^. Thus, under sulfur treatment, metabolic flux is prone to Thr synthesis for the subsequent Gly synthesis rather than Met synthesis, as reflected by the differences in the free Met content between the two treatments during grain-filling, i.e., much higher in S30 than in S0 at 7 DPA, but becomes less in S30 than in S0 after 7 DPA until grain maturity (Fig. 5b). The free Gly content in S30 is higher than in S0 at the beginning of grain-filling from 7 to 14 DPA, while it continues to decrease, making its residue in mature grain under S30 less than that of S0 (Fig. 5e). Gly is a member of aspartate-family amino acids, and its content difference between glutenins and gliadins marks the major difference between the two gluten subtypes, being 13.33% in glutenins and 1.75% in gliadins^14,18^. Thus, a higher free Gly content caused by sulfur treatment is more advantageous to glutenin biosynthesis but is less favourable for gliadin biosynthesis (Figs. 5e and 6-P6).

## Discussion

In the current study, we report the multi-faceted positive impacts of sulfur fertilisation on agronomically important traits and grain quality related traits in wheat. The study highlights the importance of soil sulfur levels in modern farming systems. N fertilisation increases both protein yield and grain yield, but its efficiency is reduced in sulfur deficient soils. Sulfur application can increase NUE-PY and NUE-GY through regulating *GS1* during plant growth and grain development, resulting in protein and grain yield increases with reduced nitrogen fertiliser addition. For grain quality, N fertilisation mainly increases the total grain protein content with a limited effect on altering the grain protein composition while sulfur fertilisation not only has a positive effect on both grain and protein yield through enhanced NUE, it also leads to optimised gluten composition for both human consumption and end-product use. An appropriate amount of sulfur application substantially decreases gliadin levels, and may subsequently reduce the incidence of wheat-related human pathologies and improve breadmaking quality.

The results of this study show that, in contrast to the negative impacts on NUE caused by increased N application^46^, sulfur application can increase NUE thus maintaining the N effects in modern wheat production systems. The preliminary comparative transcriptome analyses identified three DEGs annotated as *GS1* were related to N metabolism^40^. As a pivotal enzyme in the GOGAT cycle for N assimilation, GS can be regulated by sulfur availability, suggesting that GS links sulfur and N metabolism. During grain development, N remobilises from the leaves to the developing grains in the form of Gln, Asn, Glu and Asp^7^. Gln and Asp act as the substrates for the biosynthesis of other amino acids by catalysing transaminase activity^7^. In this study, GS activity in flag leaf was lower with sulfur addition than in the control, but the opposite was true in developing grain (Fig. 2a–d). Under sulfur treatment, the lower GS activity in flag leaf reduces the quantity of free Gln available for subsequent remobilisation to developing grains. As a consequence of this reduced GS activity, there is less free Gln available in developing grain during the early stages of grain-filling, which causes the GS in developing grain to produce more free Gln for the biosynthesis of other amino acids. Therefore, the increase in GS activity during grain-filling in response to sulfur addition indicates that sulfur application promote the participation of free Gln in developing grain, as reflected both by the sharp decrease in free Gln content in sulfur-treated plants during 7 to 21 DPA and by the differences in the dynamics of free Glu content in sulfur-treated and control plants during grain development (Fig. 2e, f). Sulfur supplementation resulted in an increase in free Glu content from 7 to 14 DPA and the rate of the subsequent decrease from 14 to 42 DPA was also greater, indicating that sulfur application facilitate the participation of free Glu in grain development (Fig. 2f). As a result, sulfur application is speculated to enhance N remobilisation from source to sink and thereby increases NUE.

Wheat gluten consists of glutenins and gliadins. High glutenin content in the wheat flour results in improved breadmaking quality^12^. The sulfur application did not exert a substantial impact on the total gluten content, but statistically significant changes in the gluten compositions were observed in samples from the 2014 field trial with all cultivars under normal N application (25 kg ha^−1^) (*P* < 0.05, *T*-test, Supplementary Data 1 and Table 3, UNIANOVA). The proportion of gliadins and each gliadin subtype in the total gluten content were significantly reduced at S30 (*P* < 0.001 at S30, *T*-test, Supplementary Data 1 and Fig. 3). Among them, ω-gliadins showed up to a 31.4% of reduction in the total gluten content, being the highest among all subtypes. The ω5-gliadins that are known to result in WDEIA were lowered by 83.9% at S50 and by 37.5% at S30 in the total gluten content. The ω1,2-gliadins, α/β-gliadins and γ-gliadins that are known to lead to wheat gluten related human disease were reduced by 25.9%, 24.2% and 19.2%, respectively, in the total gluten content at S30. Interestingly, the highest gliadin reduction occurred at S30, while the highest reduction of ω5-gliadins occurred at S50. For the gluten compositions, the overall gliadin percentage was reduced significantly while the glutenin percentage was increased by 86.7%, indicating an enhanced breadmaking quality (*P* < 0.001 at S30 and S50, *T*-test, Supplementary Data 1 and Fig. 3b).

The 2014 field trial results were confirmed in the 2014 glasshouse experiment that used 25 kg ha^−1^ N application (Table 3; Supplementary Data 1 and Supplementary Fig. 7). The trends to an improved NUE and an increased glutenin to gliadin ratio caused by sulfur supplementation at 30 kg ha^−1^ was also observed in the preliminary analysis result of 2015 glasshouse experiment which used 50 kg ha^−1^ N application; whereas the similar changes in the gliadin subtype contents caused by sulfur supplementation were not observed^40^. This may due to the excessive N availability in the pot that masked the sulfur effects in modifying each gliadin subtype content. It has been reported that either an N to sulfur ratio in the grain of more than 17 to 1 or a sulfur concentration in the grain of less than 0.2% indicates a sulfur deficient wheat grain^47^ as plants tend to maintain a relatively constant ratio of sulfur and N^48^.

This study revealed a sulfur-mediated regulatory mechanism for gluten component biosynthesis, which was verified through multiple biological assays. Several sets of high-resolution, spatial, and temporal transcriptome data generated from both Arabidopsis and cereal seed compartments provided insights into hormonal and transcription factor networks^49–51^. These data substantiate the pivotal roles of hormones and their crosstalk during seed development^52–54^. As the embryo enters the maturation phase, the concentration of ABA increases in seeds^55^. ABA is required for the induction of some seed storage protein gene expression^56,57^. In addition, the grain-filling rate in wheat is increased as a result of a higher ABA to ethylene ratio^58^. Therefore, the 416 DEGs with 18 ABA responsive *cis*-acting elements in their promoter region and the 264 DEGs encoding the corresponding transcription factors were integrated for gene interaction network construction to identify the sulfur-mediated molecular mechanism for seed storage protein biosynthesis. A study conducted on einkorn (*Triticum monococcum*, 2*n* = 14, AA) by Bonnot et al. reported that the HMT on genome A was upregulated by a sulfur deficiency treatment in glasshouse experiment^59^. However, this does not necessarily contradict our study. The HMT-1 (*TraesCS4D02G242100*) identified in our study is located on chromosome 4D, indicating a different gene locus. Furthermore, the N and sulfur fertiliser in our glasshouse experiment was mixed with the soil delivered from the field trial site before planting the germinated seeds in the pot, while Bonnot et al. supplied the N and sulfur at the stage of post-anthesis, which may result in different fertiliser impacts^59^.

A hypothesis on the potential binding between the transcription factor MYB (*TraesCS4A02G124900*) and the promoter of HMT-1 (*TraesCS4D02G242100*) were inferred from the ABA responsive gene interaction network in our study (Supplementary Figs. 4 and 6), indicating sulfur may facilitate MYB acting as a synergistic activator of HMT-1 expression to mediate the conversion of SMM to Met and then to SAM. When the SAM synthesis is accelerated, it sends signals back to mediate the upper pathways of the aspartate-family amino acid biosynthesis to reduce more SAM being synthesised^45^. As a result, more Gly is synthesised, which is less favourable for gliadin biosynthesis and accumulation (Fig. 6). This concludes that the main mechanism of sulfur-regulated gluten component biosynthesis is based on the difference in Gly content between glutenins and gliadins. Meanwhile, SAM is associated with secondary metabolite biosynthesis, which is related to grain yield formation^42^ (Fig. 6-P3). This indicates that sulfur may also insert its impact on yield through the SAM pathways apart from mediating the *GS1* activity. Also, SAM may potentially serve as a bridge in partially facilitating sulfur’s effects on grain yield and gluten components. In addition to Met, Cys is another important sulfur containing amino acid^9^. The differences in the distribution of cysteine residues among gluten components categorise them into sulfur-poor, HMW-GS, and sulfur-rich subunits. Among the three gliadin subtypes, ω-gliadins is considered as a sulfur-poor subunit^12^. Thus, sulfur treatment is adverse to ω-gliadin biosynthesis, resulting in it having the largest reduction in the mature grain. The impacts of sulfur on the formation of inter-molecular/intra-molecular disulfide bonds between two cysteine residues for gluten polymerisation are hard to reveal from the differences in the dynamics of free Cys between S0 and S30 (Fig. 5f), while the preliminary analysis result of 2015 glasshouse experiment showed that the ratio of HMW-GS to LMW-GS was decreased by sulfur supplementation^40^, thus, it can be speculated that sulfur supplementation may be prone to increase the amount of LMW-GS within glutenin components.

A study conducted on einkorn by Bonnot et al.^60^ reported that both α/β-gliadins and γ-gliadins content were increased by post-anthesis sulfur supply in a glasshouse experiment. Even though a totally different fertiliser regime was applied, the result is partly consistent with our study in that we found that the overall gliadin content was increased in S50 compared to S30 except for the ω5-gliadin content. Accordingly, the sulfur fertiliser level is related to the reduction of gliadin content. At S30, the reduction is highly remarkable while at S50 it becomes less or reversed. This implies that high levels of sulfur may cause a soil acidification problem thus masking its effects on directing gluten component biosynthesis. The soil acidification problem caused by sulfur has been a long-time research focus. It has only until recently that the impacts of sulfur deficiency on crop production has become a focus. Our results indicate that the appropriate level of sulfur fertilisation needs to be determined by measuring the existing soil sulfur level in order to achieve the effects on gliadin reduction. The result of the current study with 25 kg ha^−1^ N application suggests that 30 kg ha^−1^ sulfur application in sulfur deficiency soil can lead to less ω1,2-gliadin, α/β-gliadin and γ-gliadin content; while under high sulfur application of 50 kg ha^−1^, the WDEIA related ω5-gliadin showed the largest reduction together with an improved agronomic trait performance (PY, GY, NUE-PY and NUE-GY).

The recent rise in consumers reporting gluten avoidance has become major threat to the sustainability of the wheat industry^61^. In the past few decades, the soil in farming systems has experienced a sharp decrease in soil sulfur availability because of lower atmospheric depositions of sulfur due to cleaner fossil fuels^36^ and increased sulfur removal^37^. Our results suggest that, apart from other factors^62^, the sulfur deficiency may also contribute to the negative health aspects associated with the consumption of gluten. A range of genetic approaches have been used to reduce the level of “toxic” gliadins, however, none of the technologies allows for the safe consumption of gluten without limitations^27–32^. Moreover, the public acceptance of the end-products produced using genetic modification is still low^63^. Based on our study, a simple and straight forward approach of adopting appropriate sulfur fertiliser regimes under the growers adopted level of N usage is an alternative approach to reduce the “toxic” gliadin level.

Here we report that an appropriate sulfur supplementation under normal N application can substantially improve wheat NUE and reduce gliadin content; the associated regulatory mechanisms for wheat NUE formation and gluten component biosynthesis are also revealed. Under sulfur application condition, such mechanisms promote the increase of protein yield and grain yield and a reduction of wheat gliadin content with reduced nitrogen fertiliser input. The glutamine synthetase activity was shown to be increased by sulfur application, which result in an enhanced nitrogen source-to-sink remobilisation and assimilation and thereby an increased wheat NUE. Under grower adopted levels of nitrogen application, an appropriate level of sulfur application facilitates the biosynthesis of aspartate-family amino acids to direct gluten component biosynthesis with less gliadin but more glutenin synthesised, thereby reducing gliadin content. In summary, sulfur fertilisation has multi-faceted positive effects in sulfur deficient farming systems including yield increases, improved processing quality, and enhanced health benefits of consumers.

## Methods

### Plant material, growth conditions, and sample collection

#### Field trial

The field trial was conducted in Katanning research station of Department of Primary Industries and Regional Development (33°41′27″S, 117°33′19″E), which is located in the Western Australia Wheat Belt region in 2014. Four Australian bread wheat cultivars Livingston, Mace, Westonia and Wyalkatchem were planted with three sulfur fertilisation regimes. The soil sulfur of the trial site was measured at 4.0 mg kg^−1^ (the optimal level for crop growth is 9–10 mg kg^−1^). The fertiliser treatments consisted of three levels of sulfur fertiliser including 0, 30 and 50 kg ha^−1^ with three biologically independent replicates (or three plots) for each, expressed as S0, S30 and S50. Gypsum (18% S) was used as source of sulfur. The amount of gypsum was calculated as required in each treatment design. Urea (46% N), triple superphosphate (20.5% P) and muriate of potash (50% K) were respectively used as the sources of nitrogen, phosphorus and potassium at 25, 60 and 100 kg ha^−1^. All fertilisers were applied before sowing. The plot size was 10 m × 1.54 m = 15.4 m^2^. All plots were harvested at maturity. The NUE is determined by the GY or PY produced by per kg of nitrogen applied, calculated as the GY or PY divided by the amount of N applied at 25 kg ha^−1^, thus, the NUE-GY and NUE-PY are referred to the NUE calculated based on GY and PY, respectively.

#### Glasshouse experiment

Seeds of bread wheat cultivar Spitfire and Wyalkatchem were germinated in petri dish, and then transplanted in the 24.4 L cubic pots filled with soil (soil sulfur concentration at 4.0 mg kg^−1^) delivered from the Katanning farmland where the field trial was conducted. The experimental treatments consisted of three levels of sulfur (S0, S30, S50) with three biologically independent replicates (or three pots) for each. Same as the field trial, gypsum was used as source of sulfur and the amount was calculated as required in each treatment design; the compositions of other three basal fertiliser mixture were urea at 25 kg ha^−1^ in 2014 and at 50 kg ha^−1^ in 2015, triple superphosphate at 60 kg ha^−1^ and muriate of potash at 100 kg ha^−1^ in 2 years. All fertiliser compositions were mixed with soil before transplanting the germinated seeds in the pots. The pots were placed based on randomised complete block design. Growth conditions in glasshouse were 20/11 °C (day/night) for a 16 h light and 8 h dark photoperiod. Soil moisture was adjusted to 70% field capacity. Each pot was watered every morning with demineralised water. The flag leaf samples (1/3 of the entire flag leaf) were collected at the stage of flowering, and the main tiller developing grain samples were collected at 7-day intervals, with the developing grain sample collection starting at 7 days post-anthesis (DPA) and ending at 42 DPA. Two or three developing grains on the middle row of the main tiller head were taken, and immediately frozen in liquid nitrogen, and then stored at −80 °C. The peduncle was collected, and the mature grain was harvested. The NUE is determined by the GY produced by per kg of nitrogen applied, calculated as the GY divided by the amount of N applied at 25 kg ha^−1^, thus, the NUE-GY is referred to the NUE calculated based on GY.

### Protein extraction, separation and quantification

#### Extraction of gliadins and glutenins

The gluten components including gliadins and glutenins were sequentially extracted according to Yu et al.^64^. A 100 mg of mature grain of five cultivars from three biologically independent replicates (three pots or plots) in 2014 glasshouse experiment and 2014 field trial was respectively ground into wholemeal flour using TissueLyser II (Qiagen). A 1 ml of 70% ethanol was added to the flour for gliadin extraction. After incubating at the room temperature for 1 h and removing the supernatant as gliadins, a 1 ml of 55% isopropanol was added to remove albumins and globulins. Subsequently, 1 M 2-amino-2-(hydroxymethyl)-1,3-propanediol (TRIS)-HCl pH 8.0 and 1% DTT were added to the precipitation to disrupt the disulfide bonds, and then 1.4% 4-vinylpyridine was added to prevent the formation of reductive disulfide bonds. Afterwards, the glutenins were precipitated with 60% cold acetone. The extract was purified with 100% ethanol and acetone containing 0.07% β-mercaptoethanol, and then kept in a solution containing 0.05% trifluoroacetic acid (TFA, HPLC grade) and 50% acetonitrile (ACN, HPLC grade) before loading into the high performance liquid chromatograph (HPLC).

#### Reverse-phase high performance liquid chromatograph

The glutenins and gliadins were respectively quantified by the reverse-phase HPLC (RP-HPLC; Agilent 1200 LC system, Agilent Technologies, http://www.agilent.com). Ten microliters of the extracts were injected into a C18 reversed-phase Zorbax 300 StableBond column (4.6 × 250 mm, 5 µm, 300 Å, Agilent Technologies). The eluents were ultrapure water (solvent A) and ACN (HPLC grade, solvent B) with 0.06% TFA (HPLC grade) for each. Flow rate of 0.6 ml min^−1^ and column temperature of 60 °C were set during the running. The column was balanced for 15 min after each run. A linear gradient from 21 to 47% of solvent B in 45 min was set for glutenin or gliadin component separation. The separation profile was detected by UV absorbance at 214 nm.

#### The quantification of gliadins and glutenins

The profiles were analyzed by ChemStation LC 3D systems software (Revision B.03.02 [341], Agilent Technologies). For gliadin chromatogram, three obvious boundary areas were sequentially corresponded to ω-gliadins, α/β-gliadins and γ-gliadins based on their hydrophobicity. The amount of each gliadin subtype was calculated as the area under the chromatogram trace of each gliadin subtype. For glutenin chromatogram, two obvious boundary areas were sequentially corresponded to HMW-GS and LMW-GS based on the hydrophobicity of each protein. The amount of each glutenin subunit was calculated as the area under the chromatogram trace of each glutenin subtype.

### Free amino acid extraction, separation and quantification

Free amino acid extraction was performed according to De Barber et al.^65^. Generally, developing grains from the middle row of the main tiller head of Spitfire in 2015 glasshouse experiment were used for amino acid extraction. Since 350 mg of developing grain from each grain-filling stage is needed for amino acid extraction while a single replicate pot of plants cannot provide enough developing grain samples during the early grain development stages, the grain samples from the three replicate pots of the same developing stage and sulfur treatment were pooled together following the commonly used sample subpooling strategy^66,67^ to obtain a total of 350 mg developing grain samples. The free amino acid was extracted with Mili-Q water, followed by protein removal using acetic acid. Before derivatization, the extracts was diluted with Mili-Q water, and then a 20 μl of 30 μg ml^−1^ internal standard was added. The separation was performed by RP-HPLC using above-mentioned C18 column. The acetic acid (pH 5.8, HPLC grade) and ACN (HPLC grade) were used as running buffer for separation. The separation was performed at 0.96 ml min^−1^ in 40 °C. The injection volume was 20 μl. Quantification was performed using the under-peak-area method. The amount of each amino acid was calculated according to the internal standard. Three technical repeats were applied for each pooled developing grain sample.

### Enzyme activity assay

#### Crude enzyme extraction

A 250 mg of frozen tissues from 2015 glasshouse experiment is needed for crude enzyme extraction. The flag leaf samples (1/3 of the entire flag leaf) from three replicate pots of each cultivar (Spitfire or Wyalkatchem) under the same sulfur treatment were pooled together to reach the need of 250 mg flag leaf sample for crude enzyme extraction^66,67^. This sample subpooling strategy was also followed for sampling the developing grain for crude enzyme extraction, with the developing grain taken from the middle row of the main tiller head of each cultivar (Spitfire or Wyalkatchem) to obtain a total of 250 mg developing grain samples. The flag leaf samples and the developing grain samples were respectively sliced and then homogenised with the use of mortar and pestle in the 1.2-ml extraction buffer comprised of 100 mM Tris-HCl, 10 mM MgCl_2_, 1 mM EDTA, 3 mM DTT, 0.1% Trinton-100, and 10 mM β-ME. The homogenates were centrifuged at 15,000 × *g* for 20 min at 4 °C, and the supernatant was collected as the crude enzyme.

#### Bradford soluble protein assay

Seven ranges of BSA (bovine serum albumin) standard solution mixed with 5 ml of Bradford reagent was measured at OD_595_ for constructing the standard curve. The mixture of 0.1 ml of the crude enzyme with 5 ml of Bradford reagent was used for measuring the protein content (µg g^−1^ fresh weight). The calculation formula was ((*c*/*a*) × *V*)/*W*, in which, *c* is the protein content in the standard curve equation (mg); *a* is the measured volume of crude enzyme (ml); *V* is the volume of total extraction buffer (ml); and *W* is the fresh weight of the flag leaf sample or developing grain sample (g).

#### Glutamine synthetase activity measurement

A 0.35 ml of crude enzyme was added to 0.8 ml of GS-reaction solution comprised of 100 mM Tris-base, 20 mM MgSO_4_·7H_2_O, 100 mM l-glutamate and 10 mM hydroxylamine with pH at 7.2–8.0, and incubated at 37 °C for 5 min, followed by adding 0.35-ml of 8 mM ATP to start the reaction for 30 min at 37 °C. To end the reaction, a 0.35 ml of stop solution comprised of 2% trichloroacetic acid, 3.5% FeCl_3_·6H_2_O, and 2% HCl was added. The mixture was then centrifuged for 5 min at 5000×*g* before measuring at OD_540_. The GS activity was calculated based on the formula: ((7.9 × *A* − 0.162) × *V*) /(*P* × *T*), in which, *A* is the OD_540_ nm value; 7.9 × *A* − 0.162 is the concentration of γ-gultamylhydroxmate according to the standard curve for γ-gultamylhydroxmate; *V* is the crude enzyme volume when reaction (ml); *P* is the protein content in the crude enzyme (mg ml^−1^); and *T* is the reaction time (h). Three technical repeats were applied for each pooled flag leaf and developing grain sample.

### Electrophoretic mobility shift assay

The full-length coding sequences of MYB were amplified using the primer pair MYBF (5′-ATGGATATGGCGCACGAGAG-3′) and MYBR (5′-TCACACGAACTGCGGCTGCG-3′), and then subcloned into vector pET-30 a (+). The recombinant MYB-fusion proteins were expressed in *E.coli* strain BL21 and then purified using the His-tag column (GE Healthcare). Oligonucleotide fragments from the promoter region of HMT-1 were amplified using the primer pair 204F (5′-GTACGTTATCAACTGCATGC-3′) and 204R (5′-GAAAGGAAAGGTTAGGTGAG-3′), followed by gel purification. Briefly, the oligonucleotide fragments were incubated with MYB-fusion proteins at room temperature for 60 min, and then the mixture was separated in the nondenaturing poly-acrylamide gel. The gels were stained using an EMSA kit (Invitrogen, USA). Sequentially, the nucleic acids were stained with the SYBR^®^ green at room temperature for 20 min, followed by washing with MiliQ water for 10 s, and then visualised using 254 nm UV epi-illumination. Afterwards, the gels were stained with the SYPRO^®^ Ruby at room temperature for 3 h in the dark, followed by destaining in 10% methanol and 7% acetic acid for 60-min, and then visualised using 254 nm UV epi-illumination.

### RNA isolation, sequencing and bioinformatic analysis

#### RNA isolation, real-time PCR, sequencing and differential expressed gene analysis

Developing grains from the middle row of the main tiller head of cultivar Spitfire at 7, 14 and 21 DPA in S30 and S0 treatment from three biologically independent replicates (or three pots) in 2015 glasshouse experiment were ground in liquid nitrogen with the use of mortar and pestle. Protein in the developing grain was removed using the protein extraction buffer comprised of 1 M Tris-HCl, 5 M NaCl, 10% SDS, 0.125 M EDTA and 1 M DTT. The total RNA was extracted by sequentially adding the acid phenol/chloroform/isopropanol (49:49:2), pre-chilled Trizol (Invitrogen, Carlsbad, CA) and chloroform, and then precipitated by isopropanol, followed by using Qiagen RNase-free DNase kit to remove potential genomic DNA contamination. To check the quality of the RNA sample, concentration and purity were measured by Nanodrop with RNA typically having 260/280 ratio at approximately 2.0; the degradation and potential contamination were detected by agarose gel electrophoresis.

First strand cDNA was synthesised based on the manufacturer of Revert Aid First Strand cDNA Synthesis Kit (Thermo Fisher Scientific, USA). The expression patterns of *GS1* and *GS2* from 7 to 21 DPA in two treatments were performed by Real-time PCR using the 2^−ΔΔCT^ method (Qiagen Rotor-Gene Q instrument). The primer pairs for *GS1* and *GS2* were GS1F (5′-GTGGATGCCGTGGAGAAG-3′), GS1R (5′-GCTGAAGGTGTTGATGTCG-3′); and GS2F (5′-CTCGTCCGCGTCCTTGTCCG-3′), GS2R (5′-GCCGACCTGCCCCGCACG-3′), respectively. ADP-ribosylation factor and Actin 3 were selected as reference genes. The primer pairs for ADP-ribosylation factor and Actin 3 were ADP-RFF (5′-GCTCTCCAACAACATTGCCAAC-3′), ADP-RFR (5′-GCTTCTGCCTGTCACATACGC-3′); and Actin3F (5′-ACCTTCAGTTGCCCAGCAAT-3′), Actin3R (5′-CAGAGTCGAGCACAATACCAGTTG-3′), respectively. The relative expression level of each gene was the means of three biologically independent replicates (or three pots) ± standard deviation, and two technical repeats were conducted for each gene.

RNA integrity was confirmed with an Agilent 2100 Bioanalyzer (Agilent Technologies, Palo Alto, CA). The mRNA was enriched using oligo dT beads and then fragmented randomly in fragmentation buffer, followed by cDNA synthesis using random hexamers and reverse transcriptase. After a round of purification, terminal repair, A-tailing, ligation of sequencing adaptors, size selection and PCR enrichment, the final cDNA library was load into the Illumina HiSeq 2500 for sequencing.

The sequence datasets for 18 developing grain samples were pooled in 18 data files, in total consisting of 557,488,940 raw reads. After removing 27,415,530 reads due to the adaptor contamination, containing uncertain nucleotides N more than 10% and containing more than 50% of low quality nucleotides with base quality less than 20, the following analysis was performed on 530,073,410 clean reads. The clean reads were pair-end mapped to the IWGSC Survey sequence chromosomes version 2 by HISAT 0.1.5 beta with the mismatch setting at two nucleotides at most and other parameters setting as default for the analysis^68^. HTSeq v0.6.1 was used to analyze the gene expression level^69^. The fragment per kilo base of transcript per million mapped reads (FPKM) value of 1.0 was used as the threshold for determining the gene expression^70^. The correlation between biologically independent replicate was justified by the square of Pearson correlation coefficient more than 0.8. The negative binomial general linear model in DESeq v1.10.1 was used for differential expression analysis^71,72^. The threshold of *P*-value after normalisation (*P*-adj, *q*-value) was set as ≤0.05 to filter statistically significantly differential expressed genes (DEGs). GOseq (Release 2.12) based on Wallenius non-central hyper-geometric distribution was used for Gene Ontology (GO) enrichment analysis (http://geneontology.org/)^73^. KOBAS (v2.0, http://kobas.cbi.pku.edu.cn/) was applied for Kyoto Encyclopaedia of Genes and Genomes (KEGG) pathway enrichment analysis (http://www.genome.jp/kegg/)^74,75^. GO and KEGG pathway with false discovery rate (FDR) corrected *P*-value ≤ 0.05 were considered as statistically significant enrichment.

#### *Cis*-acting element identification, gene interaction network construction of 680 DEGs and hierarchical clustering analysis of 102 DEGs

The gene ID in Traes version was converted into TGAC version using ID History Converter in Ensembl Plants (http://plants.ensembl.org/Triticum_aestivum/Tools/AssemblyConverter; Supplementary Data 9), and the 1000 bp promoter sequence was collected from Ensembl Plants Biomart (http://plants.ensembl.org/biomart/martview). The *cis*-acting elements within 1000 bp promoter region of the 1004 DEGs were identified using the multiple promoter analysis of the ‘Gene Group Analysis’ function within the online tool PlantPAN 2.0 (http://plantpan2.itps.ncku.edu.tw/)^76^. The frequency of promoters containing the transcription factor binding site (TFBS) was set as 100%.

Pheatmap package v1.0.8 in R was used for DEGs hierarchical clustering analysis^77^. The ‘Gene prioritisation based on network direct neighbourhood’ function within WheatNet^41^ was used for gene interaction network construction, and then visualised by Cytoscape (version 3.4.0)^78^ (http://www.inetbio.org/wheatnet/).

### Statistics and reproducibility

The SPSS univariate analysis of variance (UNIANOVA) in general linear model was used to calculate the *F*-values of the genotype (G), sulfur treatment (E) and G×E on each agronomic trait and gluten component. *T*-test was carried out for multiple pairwise comparison of the agronomic traits and gluten components among different sulfur treatments. Pearson test was conducted to calculate the correlation coefficient values of peduncle traits with grain yield (nitrogen-use efficiency) under three sulfur treatments. The biologically independent replicate is defined as independent plot or pot. For the 2014 field trial, a total of 36 mature grain samples were obtained and analysed from 36 plots for agronomic trait and gluten component, including four cultivars (Livingston, Mace, Westonia and Wyalkatchem), three sulfur treatments (S0, S30 and S50) and three replicate plots for each sulfur treatment. For the 2014 glasshouse experiment, the peduncle sample size and the mature grain sample size for agronomic trait and gluten component are both 18, including two cultivars (Spitfire and Wyalkatchem), three sulfur treatments (S0, S30 and S50) and three replicate pots for each sulfur treatment. The flag leaf samples and developing grain samples collected from 2015 glasshouse experiment were used for mechanism studies, including RNA-seq assay, GS activity assay and free amino acid assay. For RNA-seq assay, grain samples of Spitfire were collected from three developing grain stages (7, 14 and 21 DPA) of two sulfur treatments (S0 and S30) with three biologically independent replicates (or three pots), making it a total of 18 samples. For GS activity and free amino acid assay, a total of six flag leaf samples of each cultivar (Spitfire or Wyalkatchem) were collected from two sulfur treatments (S0 and S30) with three biologically independent replicates (or three pots) for each sulfur treatment to measure GS activity in flag leaves; a total of 30 developing grain samples of each cultivar (Spitfire or Wyalkatchem) from five grain developing stages (7, 14, 21, 28 and 35 DPA) of two sulfur treatments (S0 and S30) with three biologically independent replicates (or three pots) were used for conveying GS activity dynamics in developing grain; a total of 36 developing grain samples of Spitfire were collected from six grain developing stages (7, 14, 21, 28, 35 and 42 DPA) of two sulfur treatments (S0 and S30) with three biologically independent replicates (or 3 pots) to extract free amino acid from developing grain. The flag leaf samples for measuring GS activity as well as the developing grain samples for conveying GS activity dynamics and extracting free amino acid were subject to the above-mentioned commonly used sample subpooling strategy^66,67^, making it two pooled flag leaf samples of each cultivar for measuring GS activity in flag leaves, ten pooled developing grain samples of each cultivar for conveying GS activity dynamics in developing grains, and 12 pooled developing grain samples of Spitfire for extracting free amino acid from developing grain, followed by three technical repeats for each.

### Reporting summary

Further information on research design is available in the Nature Research Reporting Summary linked to this article.

## Supplementary information

Supplementary Information

Description of Supplementary Files

Supplementary Data 1

Supplementary Data 2

Supplementary Data 3

Supplementary Data 4

Supplementary Data 5

Supplementary Data 6

Supplementary Data 7

Supplementary Data 8

Supplementary Data 9

Supplementary Data 10

Supplementary Data 11

Reporting Summary

## Data Availability

the RNA-seq datasets generated during the current study are available in the NCBI SRA repository. The accession code for deposited data is PRJNA719174. The metadata description is presented in Supplementary Data 10. The source data for all figure items is presented in Supplementary Data 11.
