# Supplementary material for: Impact and mechanism of sulphur-deficiency on modern wheat farming nitrogen-related sustainability and gliadin content
Source: Commun Biol. 2021 Aug 6;4:945. doi: 10.1038/s42003-021-02458-7 (PMC8346565; doi:10.1038/s42003-021-02458-7)
Supplement: Supplementary file 1 — Supplementary Information [file 42003_2021_2458_MOESM1_ESM.pdf]

Supplementary Figures

Supplementary Figure 1.

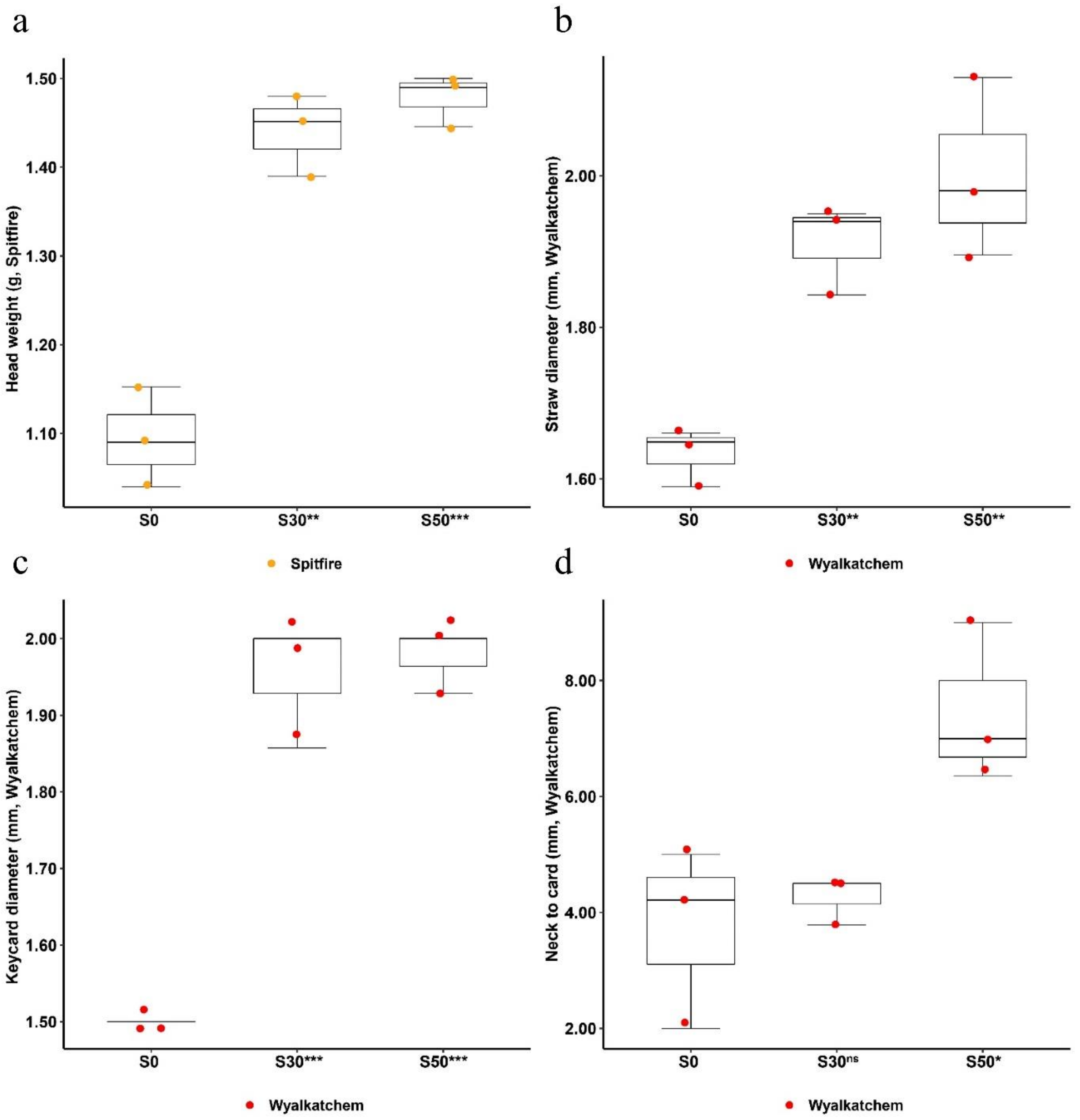

Supplementary Figure 1. Effects of sulphur treatments on peduncle traits that are statistically significantly positively correlated with grain yield (nitrogen-use efficiency) of Spitfire and Wyalkatchem in 2014 glasshouse experiment

For all panels, S0, S30, S50 mean 0, 30, and 50 kg ha^{-1} sulphur treatments, respectively. * means $P < 0.05$; ** means $P < 0.01$; *** means $P < 0.001$; ^{ns} means not significant; the number

of biologically independent replicate (or pot) for each cultivar under each sulphur treatment is 3. Boxplots show the median and interquartile ranges (IQR); the end of the top line is the third quartile (Q3) + 1.5× IQR; the end of the bottom line is the first quartile (Q1) − 1.5× IQR. The dots with orange and red respectively show the data distribution of Spitfire and Wyalkatchem and under each sulphur treatment. Panel a: The changes in head weight of Spitfire caused by S30 and S50 treatments: the head weight was increased from 1.09 g to 1.48 g; Panel b: The changes in straw diameter of Wyalkatchem caused by S30 and S50 treatments: the straw diameter was increased from 1.63 mm to 2.00 mm; Panel c: The changes in keycard diameter of Wyalkatchem caused by S30 and S50 treatments: the keycard diameter was increased from 1.50 mm to 1.98 mm; Panel d: The changes in distance between neck to card (neck to card) of Wyalkatchem caused by S30 and S50 treatments: the distance between neck to card was increased from 3.74 mm to 7.45 mm.

Supplementary Figure 2.

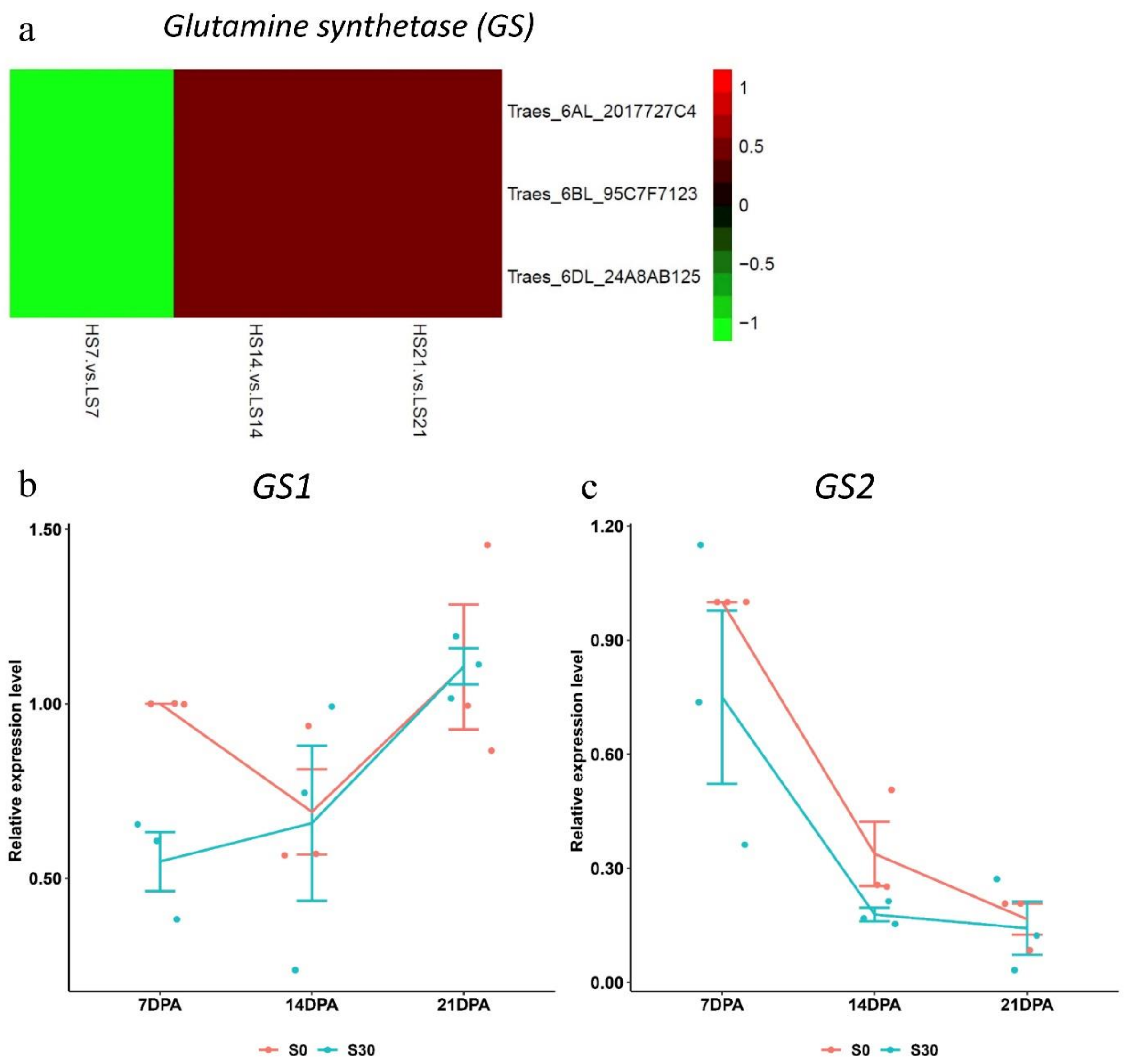

Supplementary Figure 2. The expression patterns of glutamine synthetase genes under different sulphur treatments across three grain developing stages

For all panels, DPA: days post-anthesis. Panel a: The expression patterns of the three identified DEGs in the libraries constructed based on S30 vs S0 at 7, 14, and 21 DPA: LS (S0) and HS (S30) mean 0 and 30 kg ha^{-1} sulphur treatments, respectively; DEGs: differentially expressed genes; HS7: S30 at 7 DPA; HS14: S30 at 14 DPA; HS21: S30 at 21 DPA; LS7: S0 at 7 DPA; LS14: S0 at 14 DPA; LS21: S0 at 21 DPA; the heatmap: each row represents a single gene and each column represents a library constructed between S30 vs S0 at 7, 14 and 21 DPA; the heatmap colour scale: red, green and black indicate upregulation, downregulation and no change, respectively; The three identified DEGs were *Traes_6AL_2017727C4*,

Traes_6BL_95C7F7123, and *Traes_6DL_24A8AB125*, which were all annotated as glutamine synthetase (GS). The three DEGs were all statistically significantly downregulated by S30 at 7 DPA. There was no DEGs identified in the library constructed based on S30 vs S0 at 14 and 21 DPA, indicating the differential expression of the three DEGs between S30 and S0 at 14 and 21 DPA were not statistically significant (Supplementary Data 2); Panel b: Real-time PCR analysis of the expression level of *glutamine synthetase 1* (*GS1*) under two sulphur treatments across three grain developing stages: S0 (red) and S30 (green) mean 0 and 30 kg ha^{-1} sulphur treatments, respectively; the number of biologically independent replicate (or pot) for Spitfire under each sulphur treatment is 3; line graph shows the means ± standard deviation of three biologically independent replicates (or three pots); the *GS1* was statistically significantly downregulated by S30 at 7 DPA, while the differences in the expression patterns between S30 and S0 at 14 and 21 DPA were not statistically significant; Panel c: Real-time PCR analysis of the expression level of *glutamine synthetase 2* (*GS2*) under two sulphur treatments across three grain developing stages: S0 (red) and S30 (green) mean 0 and 30 kg ha^{-1} sulphur treatments, respectively; the number of biologically independent replicate (or pot) for Spitfire under each sulphur treatment is 3; line graph shows the means ± standard deviation of three biologically independent replicates (or three pots); the differences in the expression patterns of *GS2* between S30 and S0 at 7, 14 and 21 DPA were not statistically significant.

Supplementary Figure 3.

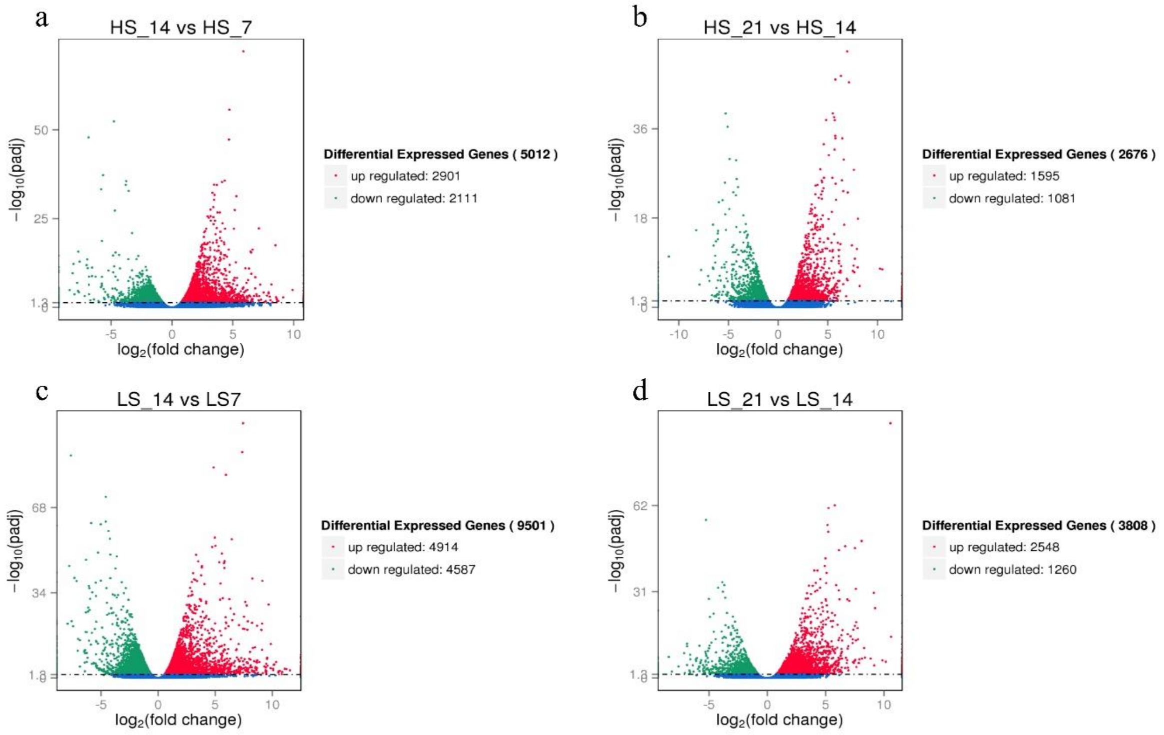

Supplementary Figure 3. The distributions of 20,997 differentially expressed genes identified among three grain developing stages of two sulphur treatments across four RNA-seq libraries

For all panels, LS (S0) and HS (S30) mean 0 and 30 kg ha^{-1} sulphur treatments, respectively; DEGs: differentially expressed genes; DPA: days post-anthesis. Panel a: The number of DEGs identified in the library constructed between 14 DPA vs 7 DPA at S30: HS_7: S30 at 7 DPA; HS_14: S30 at 14 DPA; a total of 5,012 DEGs were identified from the library of HS14 vs HS7, including 2,901 upregulated DEGs and 2,111 downregulated DEGs; Panel b: The number of DEGs identified in the library constructed between 21 DPA vs 14 DPA at S30: HS_14: S30 at 14 DPA; HS_21: S30 at 21 DPA; a total of 2,676 DEGs were identified from the library of HS21 vs HS14, including 1,595 upregulated DEGs and 1,081 downregulated DEGs; Panel c: The number of DEGs identified in the library constructed between 14 DPA vs 7 DPA at S0: LS_7: S0 at 7 DPA; LS_14: S0 at 14 DPA; a total of 9,501 DEGs were identified from the library of LS14 vs LS7, including 4,914 upregulated DEGs and 4,587 downregulated DEGs; Panel d: The number of DEGs identified in the library constructed between 21 DPA vs 14 DPA at S0: LS_14: S0 at 14 DPA; LS_21: S0 at 21 DPA; a total of 3,808 DEGs were identified from the library of LS21 vs LS14, including 2,548 upregulated DEGs and 1,260 downregulated DEGs.

Supplementary Figure 4.

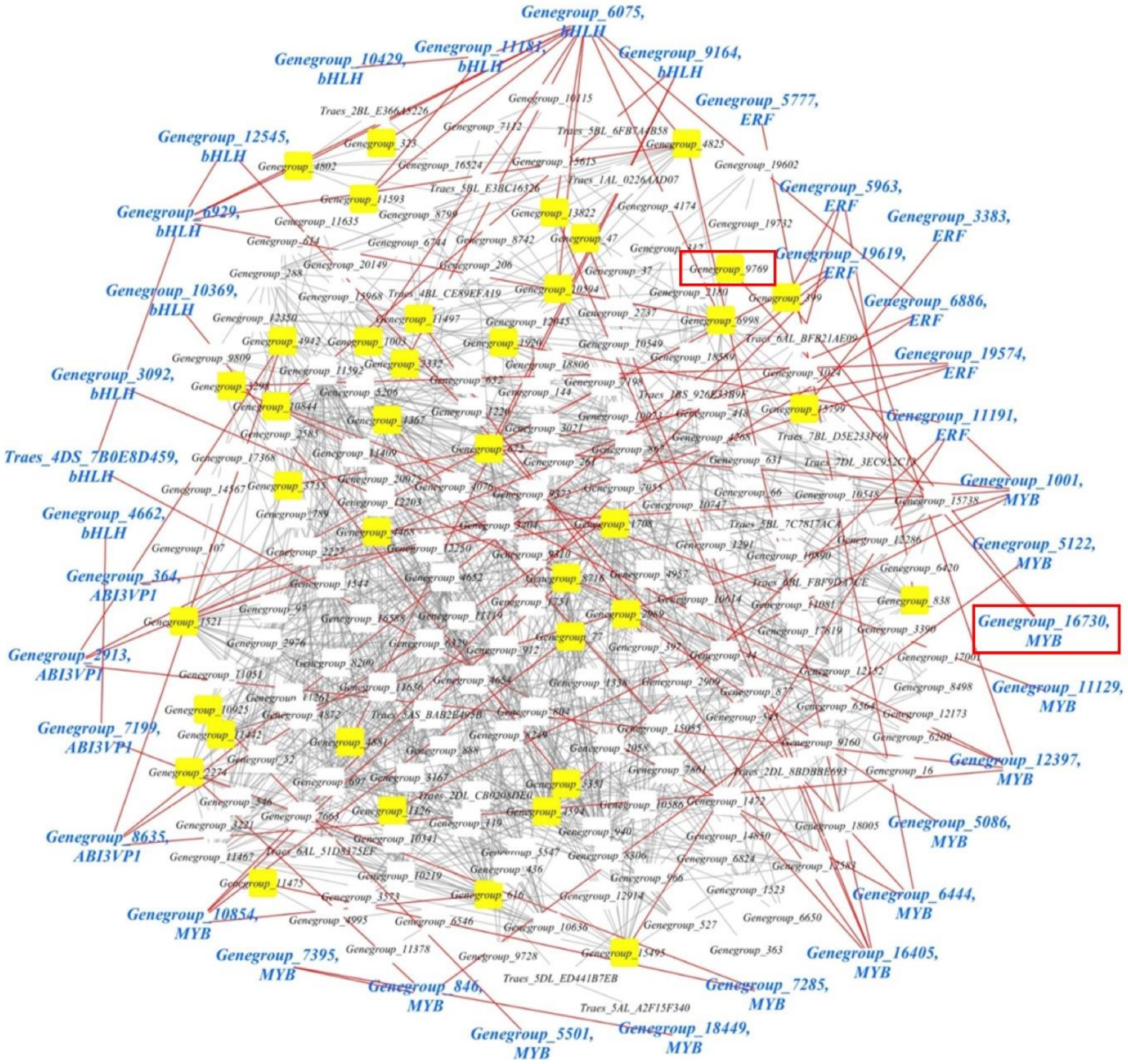

Supplementary Figure 4. ABA responsive gene interaction network.

HMT-1: homocysteine S-methyltransferase 1; blue coloured are transcription factors (52 genes); yellow labelled are the gene group (50 genes) that directly connects with the blue coloured transcription factors.; the red squares indicate the gene *TraesCS4D02G242100* annotated as HMT-1 within Genegroup_9769 and the gene *TraesCS4A02G124900* annotated as MYB within Genegroup_16730.

Supplementary Figure 5.

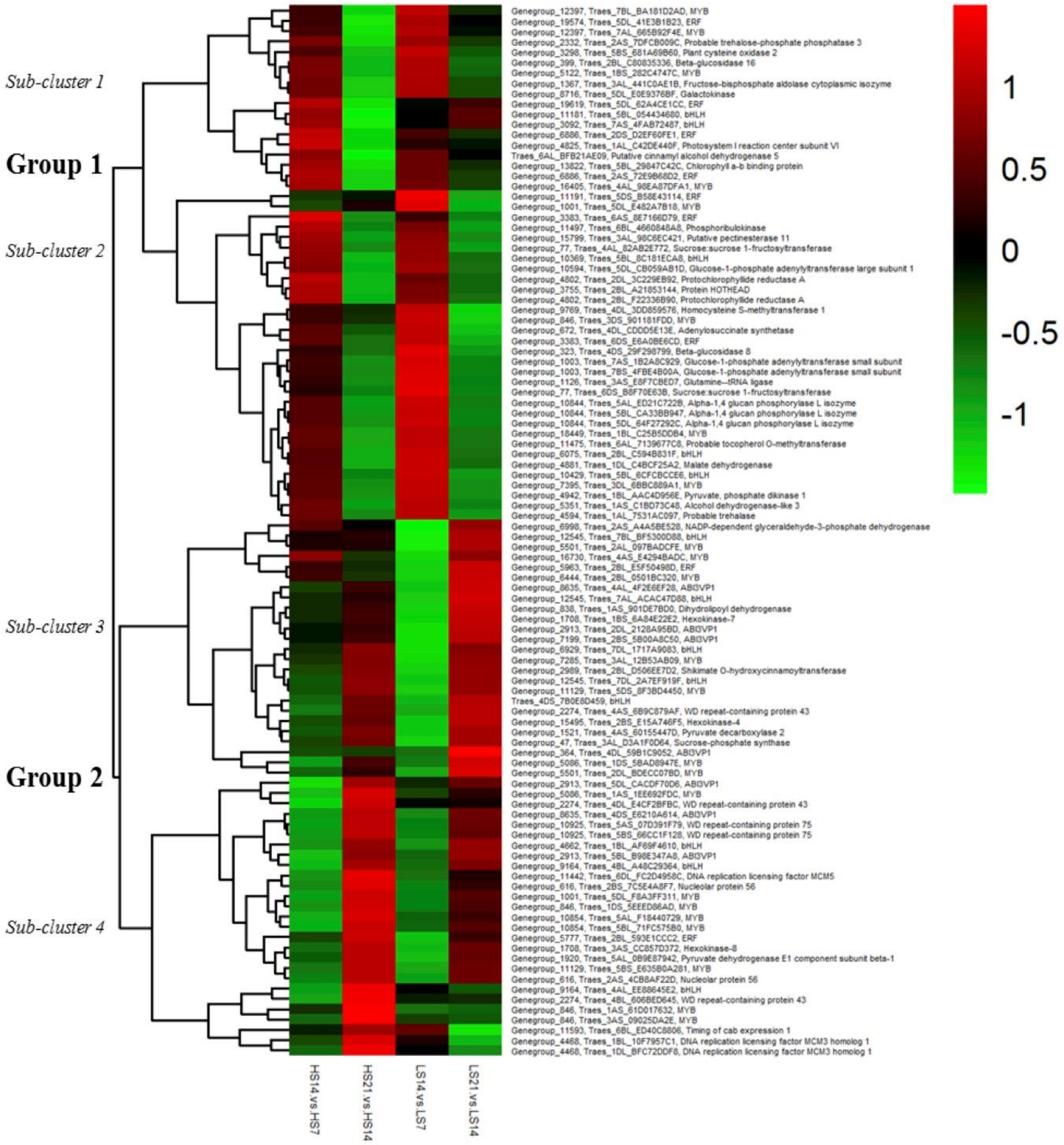

Supplementary Figure 5. Hierarchical clustering of 102 interacted genes.

LS (S0) and HS (S30) mean 0 and 30 kg ha^{-1} sulphur treatments, respectively; DPA: days post-anthesis; HS7: S30 at 7 DPA; HS14: S30 at 14 DPA; HS21: S30 at 21 DPA; LS7: S0 at 7 DPA; LS14: S0 at 14 DPA; LS21: S0 at 21 DPA; the heatmap: each row represents a single gene and its corresponding genegroup, each column represents a library constructed between two grain developing stages for S0 or S30; the heatmap colour scale: red, green and black indicate upregulation, downregulation and no change, respectively; The 102 genes were classified into

two primary clusters based on their expressed patterns across 7, 14 and 21 DPA in S0 and S30; each group was subdivided into two sub-clusters.

Supplementary Figure 6.

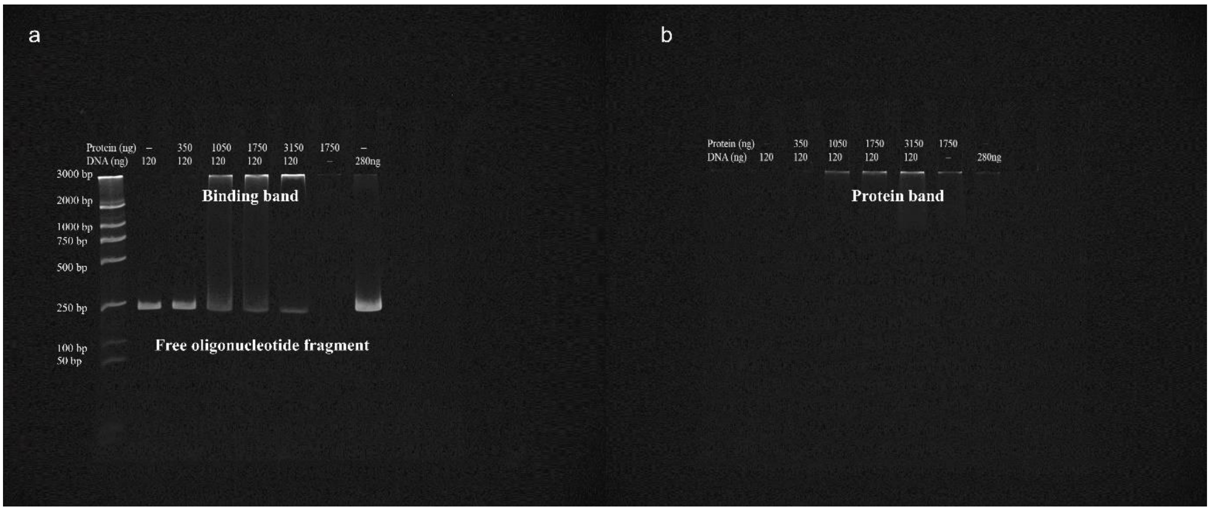

Supplementary Figure 6. Gel image of electrophoretic mobility shift assay.

Lane 1: DNA ladder; lane 2: control, blank without protein; lane 3: 350-ng protein binding with 120-ng DNA fragment; lane 4: 1050-ng protein binding with 120-ng DNA fragment; lane 5: 1750-ng protein bind with 120-ng DNA fragment; lane 6: 3150-ng protein bind with 120-ng DNA fragment; lane 7: control, blank without DNA fragment; lane 8: control, 280-ng DNA without protein; Panel a: the gels stained with SYBR® green for visualizing the DNA fragment; Panel b: the gels stained with SYPRO® Ruby for visualizing the protein band.

Supplementary Figure 7.

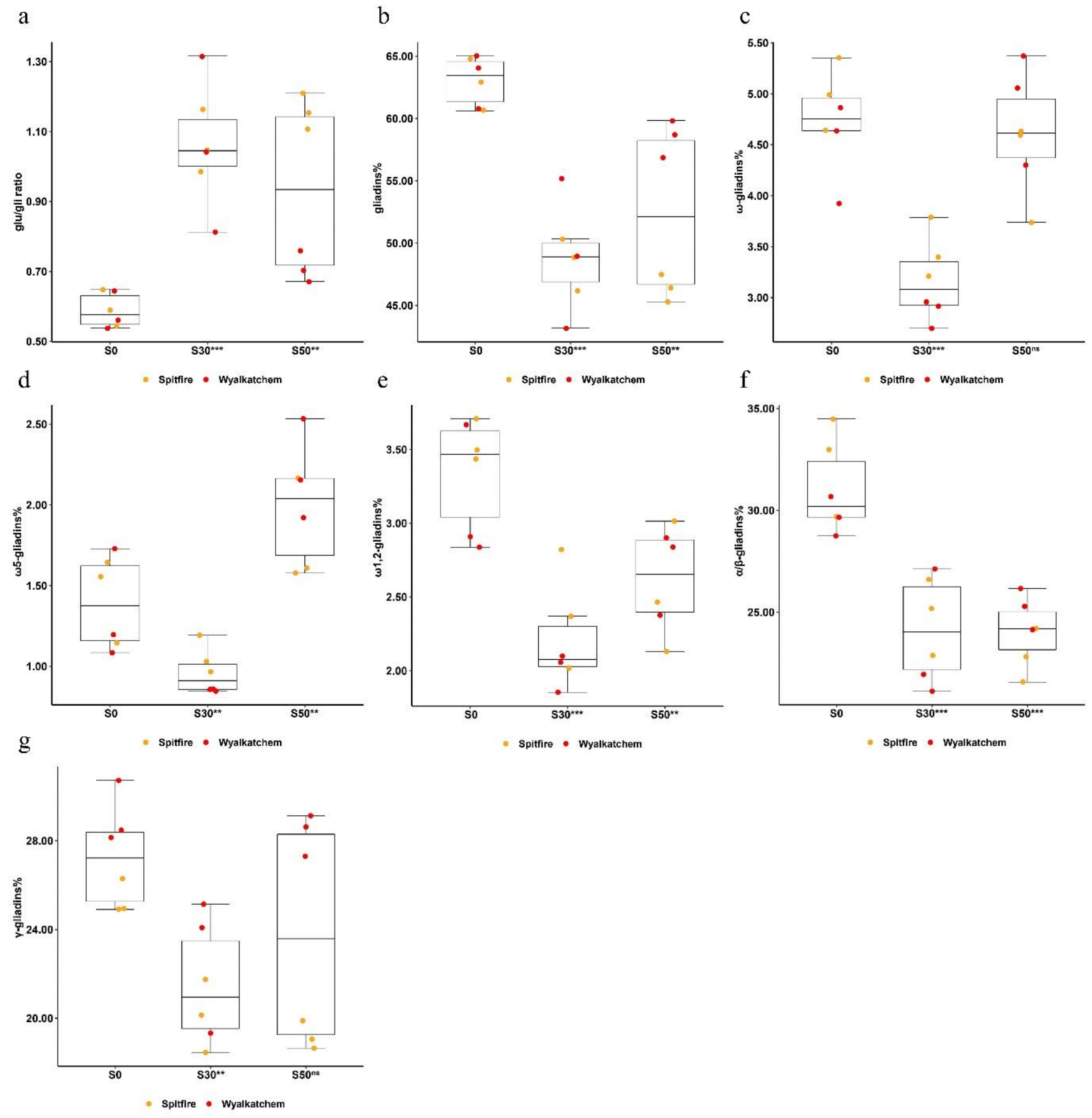

Supplementary Figure 7. Effects of sulphur treatments on the ratio of glutenin to gliadin, the percentages of gliadins and its each subtype in the total gluten content in 2014 glasshouse experiment.

For all panels, S0, S30, S50 mean 0, 30, and 50 kg ha^{-1} sulphur treatments, respectively; * means $P < 0.05$; ** means $P < 0.01$; *** means $P < 0.001$; ^{ns} means not significant; the number of biologically independent replicate (or pot) for each cultivar under each sulphur treatment is 3. Boxplots show the median and interquartile ranges (IQR); the end of the top line is the third quartile (Q3) + 1.5× IQR; the end of the bottom line is the first quartile (Q1) − 1.5× IQR. The

dots with orange and red respectively show the data distribution of Spitfire and Wyalkatchem under each sulphur treatment. Panel a: The changes in glu/gli ratio of Spitfire and Wyalkatchem caused by S30 and S50 treatments: S30 increased the average glu/gli ratio of Spitfire and Wyalkatchem from 0.59 to 1.07 and from 0.58 to 1.06, respectively; at S50, the average glu/gli ratio was increased into 1.16 for Spitfire but was reversed to 0.71 for Wyalkatchem; Panel b: The changes in the percentage of gliadins in the total gluten content (gliadins%) of Spitfire and Wyalkatchem caused by S30 and S50 treatments: S30 decreased the average gliadins% of the two cultivars from 62.8% to 48.5% and from 63.3% to 49.1%, respectively; at S50, the average gliadins% was decreased into 46.4% for Spitfire but was reversed to 58.5% for Wyalkatchem; Panel c: The changes in the percentage of ω-gliadins in the total gluten content (ω-gliadins%) of Spitfire and Wyalkatchem caused by S30 and S50 treatments: the average ω-gliadins% of the two cultivars were lowered by S30 from 4.99% to 3.47% and from 4.47% to 2.86%, respectively; at S50, the average ω-gliadins% was reversed to 4.32% and 4.91%, respectively; Panel d: The changes in the percentage of ω5-gliadins in the total gluten content (ω5-gliadins%) of Spitfire and Wyalkatchem caused by S30 and S50 treatments; the average ω5-gliadins% of the two cultivars were reduced by S30 from 1.45% to 1.06% and from 1.34% to 0.85%, respectively; at S50, the average ω5-gliadins% was reversed to 1.78% and 2.20%, respectively; Panel e: The changes in the percentage of ω1,2-gliadins in the total gluten content (ω1,2-gliadins%) of Spitfire and Wyalkatchem caused by S30 and S50 treatments: the average ω1,2-gliadins% of the two cultivars were decreased by S30 from 3.55% to 2.40% and 3.14% to 2.00%, respectively; at S50, the average ω1,2-gliadins% was reversed to 2.54% and 2.70%, respectively; Panel f: The changes in the percentage of α/β-gliadins in the total gluten content (α/β-gliadins%) of Spitfire and Wyalkatchem caused by S30 and S50 treatments: the average α/β-gliadins% of the two cultivars were reduced by S30 from 32.4% to 24.9% and from 29.7% to 23.4%, respectively; at S50, the average α/β-gliadins% of Spitfire was decreased into 22.9% for Spitfire but was reversed to 25.2% for Wyalkatchem; Panel g: The changes in the percentage of γ-gliadins in the total gluten content (γ-gliadins%) of Spitfire and Wyalkatchem caused by S30 and S50 treatments: the average γ-gliadins% of the two cultivars were reduced by S30 from 25.4% to 20.1% and from 29.1% to 22.9%, respectively; at S50, the average γ-gliadins% was decreased into 19.2% for Spitfire but reversed to 28.3% for Wyalkatchem.

Supplementary Figure 8.

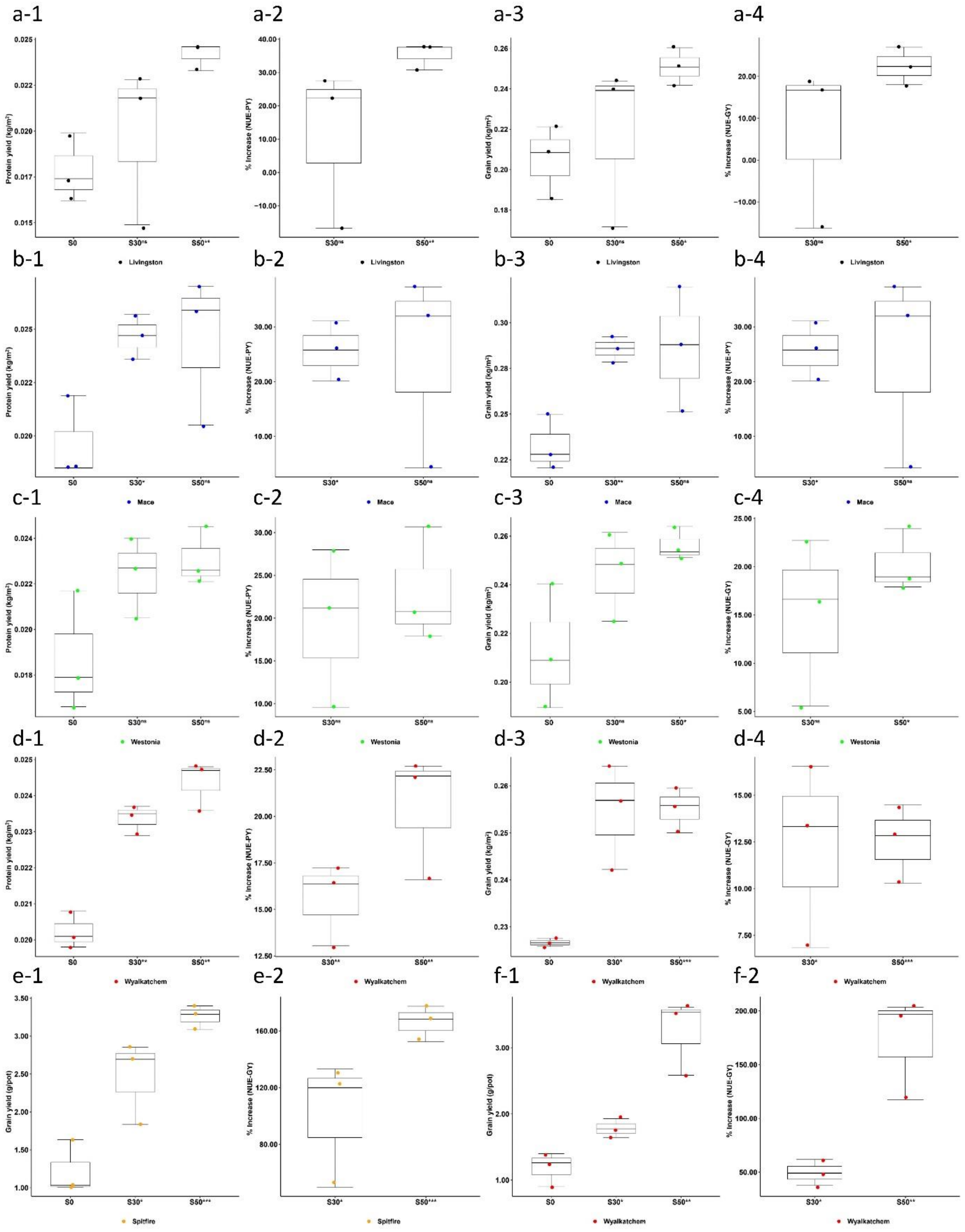

Supplementary Figure 8. Effects of sulphur treatments on protein yield, grain yield and their corresponding nitrogen-use efficiency improvement of each cultivar in 2014 field trial and 2014 glasshouse experiment.

For all panels, S0, S30, S50 mean 0, 30, and 50 kg ha^{-1} sulphur treatments, respectively. * means $P < 0.05$; ** means $P < 0.01$; *** means $P < 0.001$; ^{ns} means not significant; the number of biologically independent replicate (plot or pot) for each cultivar under each sulphur treatment is 3. Boxplots show the median and interquartile ranges (IQR); the end of the top line is the third quartile (Q3) + 1.5× IQR; the end of the bottom line is the first quartile (Q1) − 1.5× IQR. The dots with black, blue, green, red and orange respectively show the data distribution of cultivar Livingston, Mace, Westonia, Wyalkatchem and Spitfire under each sulphur treatment. Panels a-1, b-1, c-1 and d-1: The increase in the PY of cultivar Livingston, Mace, Westonia and Wyalkatchem caused by S30 and S50 treatments in 2014 field trial; Panels a-2, b-2, c-2 and d-2: The percentage of NUE-PY increase for cultivar Livingston, Mace, Westonia and Wyalkatchem at S30 and S50 treatments in 2014 field trial; Panels a-3, b-3, c-3, d-3, e-1 and f-1: The increase in the GY of cultivar Livingston, Mace, Westonia and Wyalkatchem caused by S30 and S50 treatments in 2014 field trial and cultivar Spitfire and Wyalkatchem in 2014 glasshouse experiment; Panels a-4, b-4, c-4, d-4, e-2 and f-2: The percentage of NUE-GY increase for cultivar Livingston, Mace, Westonia and Wyalkatchem at S30 and S50 treatments in 2014 field trial and cultivar Spitfire and Wyalkatchem in 2014 glasshouse experiment.

Supplementary Figure 9.

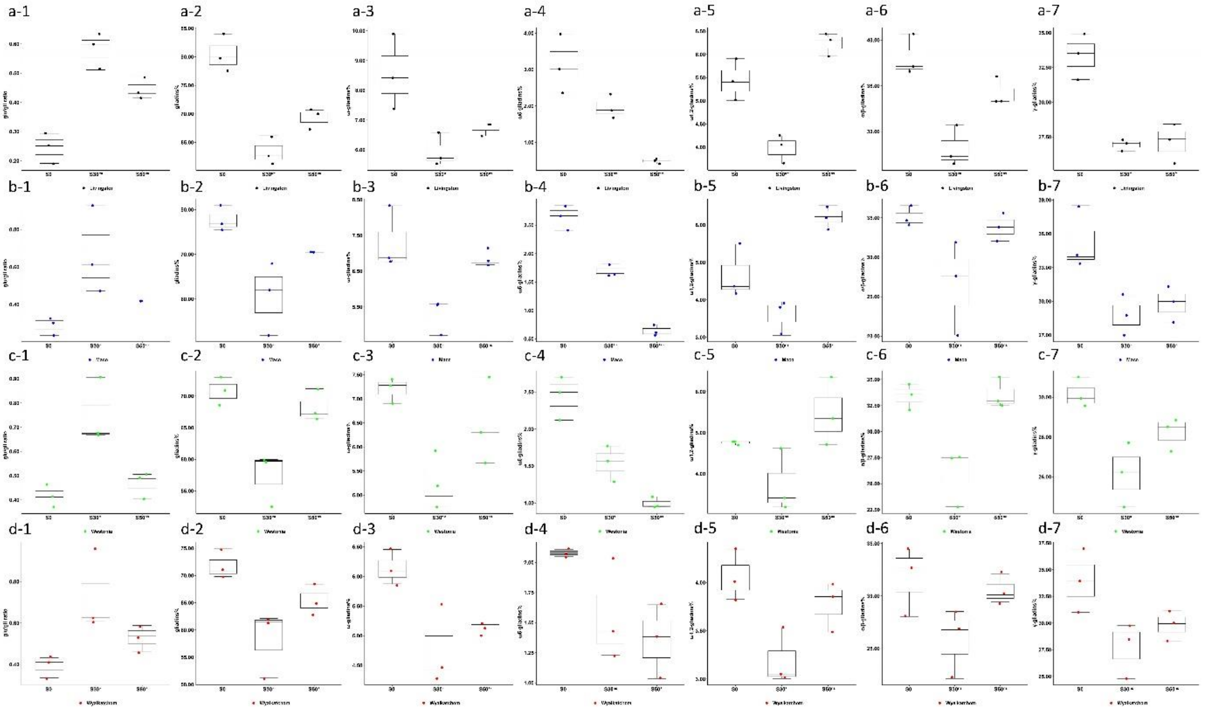

Supplementary Figure 9. Effects of sulphur treatments on the ratio of glutenin to gliadin, the percentages of gliadins and its each subtype in the total gluten content of each cultivar in 2014 field trial.

For all panels, S0, S30, S50 mean 0, 30, and 50 kg ha^{-1} sulphur treatments, respectively; * means $P < 0.05$; ** means $P < 0.01$; *** means $P < 0.001$; ^{ns} means not significant; the number of biologically independent replicate (or plot) for each cultivar under each sulphur treatment is 3. Boxplot shows the median and interquartile ranges (IQR); the end of the top line is the third quartile (Q3) + 1.5× IQR; the end of the bottom line is the first quartile (Q1) − 1.5× IQR. The dots with black, blue, green and red show the data distribution of cultivar Livingston, Mace, Westonia and Wyalkatchem under each sulphur treatment. Panels a-1, b-1, c-1 and d-1: The changes in glu/gli ratio of cultivar Livingston, Mace, Westonia and Wyalkatchem caused by S30 and S50 treatments; Panels a-2, b-2, c-2 and d-2: The changes in the percentage of gliadins in the total gluten content (gliadins%) of cultivar Livingston, Mace, Westonia and Wyalkatchem caused by S30 and S50 treatments; Panels a-3, b-3, c-3 and d-3: The changes in the percentage of ω-gliadins in the total gluten content (ω-gliadins%) of cultivar Livingston, Mace, Westonia and Wyalkatchem caused by S30 and S50 treatments; Panels a-4, b-4, c-4 and d-4: The changes in the percentage of ω5-gliadins in the total gluten content (ω5-gliadins%) of cultivar Livingston, Mace, Westonia and Wyalkatchem caused by S30 and S50 treatments;

Panels a-5, b-5, c-5 and d-5: The changes in the percentage of ω1,2-gliadins in the total gluten content (ω1,2-gliadins%) of cultivar Livingston, Mace, Westonia and Wyalkatchem caused by S30 and S50 treatments; Panels a-6, b-6, c-6 and d-6: The changes in the percentage of α/β-gliadins in the total gluten content (α/β-gliadins%) of cultivar Livingston, Mace, Westonia and Wyalkatchem caused by S30 and S50 treatments; Panels a-7, b-7, c-7 and d-7: The changes in the percentage of γ-gliadins in the total gluten content (γ-gliadins%) of cultivar Livingston, Mace, Westonia and Wyalkatchem caused by S30 and S50 treatments.

Supplementary Note 1

The following is the detailed information for the dynamics of six free amino acids during grain-filling shown in Figure 5.

CoI: content index (free amino acid% × 10^4); Met: methionine; Asn: asparagine; Asp: aspartic acid; Gly: glycine; Cys: cysteine; DPA: days post-anthesis.

Panel a: the dynamics of free total amino acid CoI during grain-filling. At 7 DPA, the free total amino acid CoI at 18.1 in S30 was slightly higher than the 16.2 in S0, but its increase to 27.9 in S30 was slightly lower than to 29.6 in S0 from 7 to 14 DPA. During 14 to 42 DPA, a decrease to 5.3 occurred in S30, which was larger than the decrease observed in S0 at 11.0; Panel b: the dynamics of free Met CoI during grain-filling. At 7 DPA, the free Met CoI in S30 at 1.34 was much higher than 1.01 measured in S0. However, during 7 to 14 DPA, a decrease to 1.26 was observed at S30, whereas an increase to 1.58 occurred in S0. The free Met CoI of the two treatments showed a similar dynamic pattern from 14 to 42 DPA, but in mature grain (42 DPA), the Met residues in S30 at 0.40 was less than in S0 at 0.53; Panel c: the dynamics of free Asn CoI during grain-filling. The free Asn CoI of 0.77 in S30 was slightly lower than the 0.94 in S0 at 7 DPA, and it was nearly the same in two treatments at 35 DPA; while a high difference between the two treatments was observed during 35 to 42 DPA, with an obvious decrease from 1.33 to 0.59 at S30 but a reversed increase from 1.35 to 1.73 at S0; Panel d: the dynamics of free Asp CoI during grain-filling. No significant difference was observed between the two treatments in free Asp CoI during 7 to 28 DPA, while a slight decrease from 1.21 to 1.02 occurred to S30 but an intensive increase from 1.19 to 2.08 happened to S0 from 28 to 42 DPA; Panel e: the dynamics of free Gly CoI during grain-filling. There was no obvious difference in the free Gly CoI (free Gly% × 10^{-4}) between the two treatments during 7 to 14 DPA; while a small fluctuate occurring to S0 from 28 to 35 DPA, the free Gly CoI was continued to decrease at S30 from 1.98 at 14 DPA to 0.21 at 42 DPA, which was lower than 0.38 in S0 at 42 DPA; Panel f: the dynamics of free Cys CoI during grain-filling. At the beginning of grain-filling, the free Cys CoI at 0.15 in S30 was lower than that in S0 at 0.20, followed by a sharper decrease to 0.01 in S30 than to 0.08 in S0 from 7 to 21 DPA. Stepping into the 21 to 35 DPA, a sharp increase to 0.10 happened to S30, whereas a slight increase to 0.11 occurred to S0. During 35 to 42 DPA, a decrease to 0.03 and 0.02 was respectively observed in S30 and S0.
