# Supplementary material for: Impact and mechanism of sulphur-deficiency on modern wheat farming nitrogen-related sustainability and gliadin content
Source: Commun Biol. 2021 Aug 6;4:945. doi: 10.1038/s42003-021-02458-7 (PMC8346565; doi:10.1038/s42003-021-02458-7)
Supplement: Supplementary file 2 — Description of Supplementary Files [file 42003_2021_2458_MOESM2_ESM.pdf]

Description of additional Supplementary Files

File name: Supplementary Data 1
Description: *P* values of multiple pairwise T-test on each agronomic trait and gluten components among three sulphur treatments in the 2014 field trial and 2014 glasshouse experiment, and the mean values are marked with statistical significance of S30 and S50 in comparison with S0 on each agronomic trait and gluten component in the 2014 field trial and the 2014 glasshouse experiment, and the data range of each trait. * means $P<0.05$, ** means $P<0.01$, *** means $P<0.001$, ns means not significant.

File name: Supplementary Data 2
Description: RNA-seq data for the three DEGs annotated as glutamine synthetase. * means $P<0.05$, ** means $P<0.01$, *** means $P<0.001$; HS7: S30 at 7 DPA; LS7: S0 at 7 DPA; HS14: S30 at14 DPA; LS14: S0 at 14 DPA; HS21: S30 at 21 DPA; LS21: S0 at 21 DPA.

File name: Supplementary Data 3
Description: List of 1,004 DEGs identified from the statistically significantly enriched GO items and KEGG pathways (corrected p-value ≤ 0.05) with gene annotation.

File name: Supplementary Data 4
Description: The 1,000-bp promoter sequence before the transcription start site (ATG) of the 1,004 DEGs.

File name: Supplementary Data 5.
Description: All cis-motifs identified from the promoter sequence of the 1,004 DEGs and their classifications based on their binding with transcription factors.

File name: Supplementary Data 6
Description: The detailed information of 40 valid cis-acting elements identified from the promoter sequence of 1,004 DEGs.

File name: Supplementary Data 7
Description: The 416 DEGs with the promoter regions containing 18 ABA responsive cis-acting elements in four libraries that were specifically constructed based on grain development stages and sulphur treatment levels.

File name: Supplementary Data 8
Description: The 264 DEGs encoded the transcription factors binding with the 18 ABA responsive cis-acting elements.

File name: Supplementary Data 9
Description: Gene ID conversion.

File name: Supplementary Data 10

Description: Metadata description for the deposited RNA-seq data in NCBI SRA repository with accession code of PRJNA719174).

File name: Supplementary Data 11
Description: Source data for the figures and supplementary figures. Sheet a: Agronomic trait data for the 2014 field trial shown in Figure 1a-1d; Sheet b: Agronomic trait data for the 2014 glasshouse experiment shown in Figure 1e-1f, and Supplementary Figure 1; Sheet c: Data for free amino acid dynamics during grain development shown in Figure 2e-2f and Figure 5; Sheet d: Data for glutamine synthetase (GS) activity in flag leaf and developing grain of Spitfire and Wyalkatchem shown in Figure 2a-2d; Sheet e: Gluten component data for the 2014 field trial shown in Figure 3; Sheet f: Data for Real-time PCR to detect GS expression patterns during grain development at S30 and S0 treatments shown in Supplementary Figure 2; Sheet g: Gluten component data for the 2014 glasshouse experiment.
