# Supplementary material for: Impact and mechanism of sulphur-deficiency on modern wheat farming nitrogen-related sustainability and gliadin content
Source: Commun Biol. 2021 Aug 6;4:945. doi: 10.1038/s42003-021-02458-7 (PMC8346565; doi:10.1038/s42003-021-02458-7)
Supplement: Supplementary file 3 — Supplementary Data 1 [file 42003_2021_2458_MOESM3_ESM.pdf]

	Field trial		
	Grain yield	Protein yield	Grain yield
S30 vs S0	0.0077	0.0017	0.0034
S50 vs S0	0.0000	0.0000	0.0000
S50 vs S30	0.3180	0.1394	0.0015

Note: *P* values of multiple pairwise T-test on each agronomic trait and gluten comp

	Field trial		
	Grain yield (kg/m^2)	Protein yield (kg/m2)	Grain yield (g/pot)
S0	0.22	0.019	1.21
S30	0.25**	0.023**	2.12**
S50	0.26***	0.024***	3.25***

Note: Mean values are marked with statistical significance of S30 and S50 in compa

P <0.05	*
P <0.01	**
P <0.001	***
ns	not significant

Agronomic trait	
	Glasshouse experiment
Neck to card (Wyalkatchem)	Straw diameter (Wyalkatchem)
0.6030	0.0023
0.0364	0.0068
0.0184	0.3006

ponents among three sulphur treatments in the 2014 field trial and 2014 glasshou

Agronomic trait	
	Glasshouse experiment
Neck to card (mm, Wyalkatchem)	Straw diameter (mm, Wyalkatchem)
3.74	1.63
4.26^{ns}	1.91**
7.45*	2.00**

arison with S0 on each agronomic trait and gluten component in the 2014 field tri

Keycard diameter (Wyalkatchem)	Head weight (Spitfire)	glu/gli	gliadins%
0.0007	0.0012	0.0000	0.0000
0.0000	0.0005	0.0002	0.0003
0.6779	0.2912	0.0002	0.0001

ıse experiment.

Keycard diameter (mm, Wyalkatchem)	Head weight (g, Spitfire))	glu/gli	gliadins (%)
1.50	1.09	0.34	75.18
1.95***	1.44**	0.68***	59.90***
1.98***	1.48***	0.46***	68.36***

al and the 2014 glasshouse experiment.

Gluten composition

Field trial					
ω5-gliadins%	ω1,2-gliadins%	ω-gliadins%	α/β-gliadins%	γ-gliadins%	glu/gli
0.0001	0.0001	0.0000	0.0000	0.0000	0.0001
0.0000	0.0764	0.0204	0.2386	0.0001	0.0073
0.0000	0.0000	0.0041	0.0001	0.0703	0.3247

Gluten composition

Field trial					
ω5-gliadins (%)	ω1,2-gliadins (%)	ω-gliadins (%)	α/β-gliadins (%)	γ-gliadins (%)	glu/gli
2.57	4.74	7.31	34.68	33.19	0.59
1.69***	3.62***	5.32***	27.20***	27.38***	1.06***
0.87***	5.43^{ns}	6.30*	33.33^{ns}	28.74***	0.93**

Glasshouse expriment

gliadins%	ω5-gliadins%	ω1,2-gliadins%	ω-gliadins%	α/β-gliadins%	γ-gliadins%
0.0000	0.0068	0.0003	0.0001	0.0005	0.0025
0.0040	0.0098	0.0063	0.7018	0.0001	0.1565
0.2837	0.0001	0.0635	0.0004	0.9274	0.3511

Glasshouse expriment

gliadins (%)	ω5-gliadins (%)	ω1,2-gliadins (%)	ω-gliadins (%)	α/β-gliadins (%)	γ-gliadins (%)
63.02	1.39	3.34	4.73	31.04	27.25
48.79***	0.96**	2.20***	3.16***	24.15***	21.48**
52.42**	1.99**	2.62**	4.61^{ns}	24.03***	23.77^{ns}

Field_trial

Varname	Streat	Ntreat	range	GrainYield_(kg/m2)	ProtYield_(kg/m2)	NUE_GY
Wyalkatchem	S0	N25	smallest_value	0.2258	0.0198	90.3325
			largest_value	0.2276	0.0208	91.0260
Westonia	S0	N25	smallest_value	0.1895	0.0166	75.8156
			largest_value	0.2404	0.0217	96.1792
Livingston	S0	N25	smallest_value	0.1853	0.0162	74.1143
			largest_value	0.2211	0.0199	88.4312
Mace	S0	N25	smallest_value	0.2205	0.0185	88.1870
			largest_value	0.2499	0.0219	99.9455
Wyalkatchem	S30	N25	smallest_value	0.2422	0.0229	96.8753
			largest_value	0.2642	0.0237	105.6883
Westonia	S30	N25	smallest_value	0.2249	0.0205	89.9455
			largest_value	0.2614	0.0240	104.5584
Livingston	S30	N25	smallest_value	0.1716	0.0149	68.6494
			largest_value	0.2437	0.0228	97.4831
Mace	S30	N25	smallest_value	0.2785	0.0236	111.3896
			largest_value	0.2924	0.0257	116.9506
Wyalkatchem	S50	N25	smallest_value	0.2500	0.0236	100.0000
			largest_value	0.2595	0.0248	103.8104
Westonia	S50	N25	smallest_value	0.2511	0.0221	100.4545
			largest_value	0.2640	0.0245	105.6130
Livingston	S50	N25	smallest_value	0.2419	0.0233	96.7688
			largest_value	0.2603	0.0246	104.1221
Mace	S50	N25	smallest_value	0.2512	0.0205	100.4961
			largest_value	0.3194	0.0270	127.7792

Glasshouse_experiment

Varname	Streat	Ntreat	range	GrainYield_(g/pot)	NUE_GY	neckdiame
Spitfire	S0	N25	smallest_value	1.0060	0.4024	1.5000
			largest_value	1.6330	0.6532	1.5800
Wyalkatchem	S0	N25	smallest_value	0.9080	0.3632	1.3700
			largest_value	1.4010	0.5604	1.4900
Spitfire	S30	N25	smallest_value	1.8330	0.7332	1.5386
			largest_value	2.8550	1.1420	1.5700
Wyalkatchem	S30	N25	smallest_value	1.6410	0.6564	1.6800
			largest_value	1.9260	0.7704	1.7500
Spitfire	S50	N25	smallest_value	3.0900	1.2360	1.4100
			largest_value	3.3980	1.3592	1.4800
Wyalkatchem	S50	N25	smallest_value	2.5880	1.0352	1.6100
			largest_value	3.6120	1.4448	1.6600

NUE_PY	glu/gli	ω_5%	ω_1,2%	α/β%	γ%	ω%	gliadins%
7.9163	0.3349	0.0205	0.0383	0.2804	0.3101	0.0588	0.6981
8.3265	0.4324	0.0211	0.0435	0.3448	0.3692	0.0645	0.7491
6.6357	0.3712	0.0212	0.0470	0.3205	0.2949	0.0690	0.6845
8.6641	0.4609	0.0270	0.0478	0.3452	0.3101	0.0740	0.7293
6.4955	0.1910	0.0237	0.0500	0.3727	0.3163	0.0738	0.7731
7.9524	0.2936	0.0398	0.0591	0.4054	0.3489	0.0989	0.8396
7.3828	0.2355	0.0241	0.0421	0.3409	0.3351	0.0676	0.7545
8.7738	0.3254	0.0285	0.0549	0.3656	0.3851	0.0834	0.8094
9.1494	0.6097	0.0123	0.0300	0.2213	0.2479	0.0428	0.5120
9.4892	0.9531	0.0203	0.0353	0.2852	0.2979	0.0556	0.6212
8.2010	0.6673	0.0129	0.0319	0.2279	0.2449	0.0476	0.5246
9.5828	0.9061	0.0177	0.0462	0.2753	0.2777	0.0591	0.5998
5.9488	0.5111	0.0168	0.0364	0.2919	0.2647	0.0553	0.6137
9.1068	0.6294	0.0234	0.0424	0.3257	0.2725	0.0658	0.6618
9.4356	0.4717	0.0160	0.0305	0.2015	0.2701	0.0470	0.5186
10.2972	0.9282	0.0182	0.0395	0.3189	0.3048	0.0559	0.6795
9.4374	0.4620	0.0104	0.0349	0.2944	0.2832	0.0502	0.6300
9.9313	0.5873	0.0165	0.0399	0.3211	0.3115	0.0524	0.6840
8.8287	0.4056	0.0095	0.0472	0.3251	0.2719	0.0566	0.6642
9.7842	0.5055	0.0109	0.0636	0.3522	0.2900	0.0744	0.7115
9.3366	0.4158	0.0041	0.0596	0.3464	0.2557	0.0647	0.6707
9.8345	0.4910	0.0055	0.0644	0.3682	0.2842	0.0685	0.7063
8.1896	0.4178	0.0056	0.0593	0.3203	0.2810	0.0668	0.7046
10.7862	0.4192	0.0075	0.0652	0.3564	0.3129	0.0713	0.7053

headweig	pedunclel	strawdia	keycarddia	necktocard	strawweigh	glu/gli	ω_5%	ω_1,2%
1.0400	13.0000	1.6100	1.5000	1.0000	0.0500	0.5442	0.0115	0.0344
1.1529	19.4286	1.6471	1.5714	4.6429	0.0514	0.6495	0.0164	0.0371
0.7100	14.0000	1.5900	1.5000	2.0000	0.0500	0.5379	0.0108	0.0284
0.9300	15.5000	1.6600	1.5000	5.0000	0.0557	0.6442	0.0173	0.0367
1.3900	22.2857	1.8400	1.8571	8.0000	0.0571	0.9867	0.0097	0.0202
1.4800	25.0000	1.9600	2.0000	12.0000	0.0700	1.1634	0.0119	0.0237
1.2800	17.0000	1.8429	1.8571	3.7857	0.0500	0.8120	0.0085	0.0185
1.5500	20.0000	1.9500	2.0000	4.5000	0.0700	1.3163	0.0086	0.0210
1.4457	19.5000	1.6100	1.5000	3.0000	0.0500	1.1081	0.0158	0.0213
1.5000	21.5000	1.9400	2.0000	10.0000	0.0800	1.2100	0.0217	0.0301
1.3100	18.0000	1.8957	1.9286	6.3571	0.0543	0.6710	0.0192	0.0238
1.4800	21.5000	2.1300	2.0000	9.0000	0.0600	0.7600	0.0253	0.0290

α/β%	γ%	ω%	gliadins%
0.2970	0.2491	0.0464	0.6062
0.3450	0.2628	0.0535	0.6476
0.2875	0.2815	0.0392	0.6082
0.3067	0.3073	0.0486	0.6502
0.2288	0.1845	0.0321	0.4622
0.2660	0.2175	0.0379	0.5033
0.2113	0.1934	0.0270	0.4317
0.2713	0.2514	0.0296	0.5519
0.2156	0.1864	0.0374	0.4525
0.2420	0.1988	0.0463	0.4744
0.2415	0.2730	0.0430	0.5682
0.2617	0.2912	0.0537	0.5984
