# Supplementary material for: Impact and mechanism of sulphur-deficiency on modern wheat farming nitrogen-related sustainability and gliadin content
Source: Commun Biol. 2021 Aug 6;4:945. doi: 10.1038/s42003-021-02458-7 (PMC8346565; doi:10.1038/s42003-021-02458-7)
Supplement: Supplementary file 13 — Supplementary Data 11 [file 42003_2021_2458_MOESM13_ESM.pdf]

Varname	Streat	Ntreat	GrainYield_(kg/m2)	ProtYield_(kg/m2)	NUE_GY	NUE_PY
Wyalkatchem	S0	N25	0.2258	0.0198	90.3325	7.9163
Wyalkatchem	S0	N25	0.2276	0.0201	91.0260	8.0390
Wyalkatchem	S0	N25	0.2266	0.0208	90.6494	8.3265
Westonia	S0	N25	0.2090	0.0179	83.6182	7.1631
Westonia	S0	N25	0.2404	0.0217	96.1792	8.6641
Westonia	S0	N25	0.1895	0.0166	75.8156	6.6357
Livingston	S0	N25	0.2085	0.0174	83.3974	6.9765
Livingston	S0	N25	0.1853	0.0162	74.1143	6.4955
Livingston	S0	N25	0.2211	0.0199	88.4312	7.9524
Mace	S0	N25	0.2280	0.0185	91.2052	7.4112
Mace	S0	N25	0.2205	0.0185	88.1870	7.3828
Mace	S0	N25	0.2499	0.0219	99.9455	8.7738
Wyalkatchem	S30	N25	0.2569	0.0229	102.7506	9.1494
Wyalkatchem	S30	N25	0.2642	0.0237	105.6883	9.4892
Wyalkatchem	S30	N25	0.2422	0.0235	96.8753	9.4196
Westonia	S30	N25	0.2614	0.0240	104.5584	9.5828
Westonia	S30	N25	0.2249	0.0205	89.9455	8.2010
Westonia	S30	N25	0.2484	0.0227	99.3714	9.0725
Livingston	S30	N25	0.2437	0.0228	97.4831	9.1068
Livingston	S30	N25	0.1716	0.0149	68.6494	5.9488
Livingston	S30	N25	0.2392	0.0218	95.6649	8.7323
Mace	S30	N25	0.2924	0.0257	116.9506	10.2972
Mace	S30	N25	0.2785	0.0236	111.3896	9.4356
Mace	S30	N25	0.2860	0.0247	114.4078	9.8798
Wyalkatchem	S50	N25	0.2558	0.0236	102.3117	9.4374
Wyalkatchem	S50	N25	0.2595	0.0248	103.8104	9.9313
Wyalkatchem	S50	N25	0.2500	0.0247	100.0000	9.8892
Westonia	S50	N25	0.2511	0.0221	100.4545	8.8287
Westonia	S50	N25	0.2534	0.0226	101.3610	9.0427
Westonia	S50	N25	0.2640	0.0245	105.6130	9.7842
Livingston	S50	N25	0.2603	0.0246	104.1221	9.8274
Livingston	S50	N25	0.2419	0.0246	96.7688	9.8345
Livingston	S50	N25	0.2508	0.0233	100.3247	9.3366
Mace	S50	N25	0.3194	0.0270	127.7792	10.7862
Mace	S50	N25	0.2879	0.0259	115.1506	10.3667
Mace	S50	N25	0.2512	0.0205	100.4961	8.1896

Varname	Streat	Ntreat	GrainYield_(g/pot)	NUE_GY	neckdiameter	headweight
Spitfire	S0	N25	1.6330	0.6532	1.5043	1.1529
Spitfire	S0	N25	1.0340	0.4136	1.5000	1.0900
Spitfire	S0	N25	1.0060	0.4024	1.5800	1.0400
Wyalkatchem	S0	N25	1.4010	0.5604	1.4186	0.8471
Wyalkatchem	S0	N25	0.9080	0.3632	1.3700	0.7100
Wyalkatchem	S0	N25	1.2620	0.5048	1.4900	0.9300
Spitfire	S30	N25	2.8550	1.1420	1.5386	1.4514
Spitfire	S30	N25	2.6930	1.0772	1.5400	1.4800
Spitfire	S30	N25	1.8330	0.7332	1.5700	1.3900
Wyalkatchem	S30	N25	1.9260	0.7704	1.6943	1.3186
Wyalkatchem	S30	N25	1.6410	0.6564	1.6800	1.5500
Wyalkatchem	S30	N25	1.7750	0.7100	1.7500	1.2800
Spitfire	S50	N25	3.3980	1.3592	1.4743	1.4457
Spitfire	S50	N25	3.0900	1.2360	1.4100	1.5000
Spitfire	S50	N25	3.2860	1.3144	1.4800	1.4900
Wyalkatchem	S50	N25	3.6120	1.4448	1.6100	1.3171
Wyalkatchem	S50	N25	2.5880	1.0352	1.6400	1.4800
Wyalkatchem	S50	N25	3.5350	1.4140	1.6600	1.3100

pedunclelength	strawdiameter	keycarddiameter	necktocard	strawweight
19.4286	1.6471	1.5714	4.6429	0.0514
15.5000	1.6100	1.5000	2.0000	0.0500
13.0000	1.6200	1.5000	1.0000	0.0500
14.1429	1.6486	1.5000	4.2143	0.0557
14.0000	1.5900	1.5000	5.0000	0.0500
15.5000	1.6600	1.5000	2.0000	0.0500
22.2857	1.8400	1.8571	8.9286	0.0571
25.0000	1.8600	2.0000	12.0000	0.0700
23.0000	1.9600	2.0000	8.0000	0.0700
17.2857	1.8429	1.8571	3.7857	0.0614
17.0000	1.9400	2.0000	4.5000	0.0500
20.0000	1.9500	2.0000	4.5000	0.0700
21.5000	1.7786	1.7143	6.4286	0.0543
21.0000	1.6100	1.5000	3.0000	0.0500
19.5000	1.9400	2.0000	10.0000	0.0800
18.1429	1.8957	1.9286	6.3571	0.0543
21.5000	1.9800	2.0000	7.0000	0.0600
18.0000	2.1300	2.0000	9.0000	0.0600

Streat	DPA	REP	ASP	GLU	ASN	SER	GLN	ARG	GLY	THR	PRO	ALA
S0	7DPA	tech1	0.8669	3.3690	0.9461	1.1841	3.1922	0.7518	0.1061	0.3370	1.3643	1.9004
S0	7DPA	tech2	0.8604	3.3556	0.9370	1.1853	3.0726	0.7455	0.1128	0.3252	1.3494	1.8991
S0	7DPA	tech3	0.8649	3.3683	0.9471	1.1875	3.1249	0.7506	0.1102	0.3327	1.3518	1.9015
S0	14DPA	tech1	1.6091	6.6949	2.1035	2.5285	2.4771	0.3691	1.8091	0.7734	0.5602	8.0339
S0	14DPA	tech2	1.6090	6.6971	2.1041	2.5399	2.2955	0.3317	1.8609	0.7535	0.5590	8.0259
S0	14DPA	tech3	1.6010	6.6995	2.1045	2.5338	2.2831	0.3440	1.8381	0.7619	0.5585	8.0193
S0	21DPA	tech1	1.4182	5.6435	0.5112	1.2849	1.1590	0.1851	0.4001	0.6143	0.5508	4.6485
S0	21DPA	tech2	1.4138	5.6232	0.5109	1.2857	1.0892	0.1747	0.4292	0.5692	0.5489	4.6421
S0	21DPA	tech3	1.4133	5.6160	0.5103	1.2853	1.0812	0.1723	0.4331	0.5667	0.5482	4.6411
S0	28DPA	tech1	1.1882	4.3563	0.3798	0.7891	1.1904	0.1738	0.2419	0.4181	0.3830	3.9767
S0	28DPA	tech2	1.1858	4.3378	0.3794	0.7879	1.1069	0.1678	0.2538	0.4098	0.3836	3.9703
S0	28DPA	tech3	1.1825	4.3413	0.3792	0.7882	1.1488	0.1680	0.2496	0.4135	0.3831	3.9660
S0	35DPA	tech1	2.2229	4.4057	1.3497	0.6551	0.9039	0.9669	0.7246	0.4205	0.3553	4.3246
S0	35DPA	tech2	2.2240	4.4087	1.3477	0.6562	0.8765	0.9542	0.7377	0.4187	0.3509	4.3242
S0	35DPA	tech3	2.2208	4.4033	1.3472	0.6556	0.9145	0.9506	0.7352	0.4224	0.3508	4.3244
S0	42DPA	tech1	2.0633	1.5455	1.7143	0.3080	0.2035	0.8386	0.3678	0.2293	0.3072	2.0903
S0	42DPA	tech2	2.0863	1.5579	1.7323	0.3099	0.1638	0.8322	0.3941	0.2207	0.3073	2.1108
S0	42DPA	tech3	2.0853	1.5644	1.7336	0.3104	0.1864	0.8345	0.3869	0.2271	0.3076	2.1124
S30	7DPA	tech1	0.8336	3.5560	0.7681	1.4708	3.0318	0.6630	0.2212	0.3796	1.7002	3.2037
S30	7DPA	tech2	0.8286	3.5408	0.7672	1.4763	2.8984	0.6476	0.2491	0.3623	1.6933	3.1998
S30	7DPA	tech3	0.8288	3.5358	0.7658	1.4736	2.8980	0.6423	0.2507	0.3620	1.6929	3.1992
S30	14DPA	tech1	1.4621	7.2703	1.4722	2.8608	1.5078	0.2808	1.9312	0.8430	0.4906	7.4194
S30	14DPA	tech2	1.4622	7.2577	1.4724	2.8610	1.2424	0.2447	2.0033	0.8011	0.4890	7.4134
S30	14DPA	tech3	1.4606	7.2676	1.4719	2.8614	1.2530	0.2460	1.9915	0.8101	0.4885	7.4119
S30	21DPA	tech1	1.6772	3.8222	0.7751	1.4016	0.6603	0.2103	0.8816	0.5441	0.4920	3.5383
S30	21DPA	tech2	1.6791	3.8178	0.7761	1.3997	0.5866	0.1969	0.9087	0.4997	0.4918	3.5377
S30	21DPA	tech3	1.6770	3.8118	0.7738	1.3990	0.6077	0.1966	0.9044	0.5028	0.4881	3.5299
S30	28DPA	tech1	1.2057	5.1669	1.1770	0.8149	1.2708	0.1859	0.6312	0.4106	0.2428	5.5364
S30	28DPA	tech2	1.2070	5.1600	1.1760	0.8150	1.1870	0.1787	0.6417	0.4059	0.2426	5.5284
S30	28DPA	tech3	1.2026	5.1545	1.1737	0.8133	1.2416	0.1777	0.6390	0.4085	0.2437	5.5218
S30	35DPA	tech1	1.4425	3.2929	1.3269	0.5041	0.9254	0.5015	0.4418	0.3028	0.3214	3.2223
S30	35DPA	tech2	1.4511	3.2963	1.3289	0.5044	0.8864	0.4918	0.4548	0.2826	0.3219	3.2224
S30	35DPA	tech3	1.4483	3.2939	1.3282	0.5043	0.8891	0.4884	0.4570	0.2829	0.3214	3.2222
S30	42DPA	tech1	1.0230	0.4383	0.5918	0.1934	0.1121	0.2622	0.2029	0.1669	0.2127	1.1440
S30	42DPA	tech2	1.0243	0.4430	0.5963	0.1966	0.1068	0.2558	0.2155	0.1645	0.2144	1.1419
S30	42DPA	tech3	1.0198	0.4372	0.5914	0.1935	0.1109	0.2553	0.2109	0.1656	0.2125	1.1434

TYR	VAL	MET	CYS	ILE	LEU	PHE	AA
0.0118	0.1680	1.0102	0.2041	0.3643	0.4224	0.1263	16.3248
0.0129	0.1689	1.0135	0.2045	0.3653	0.4238	0.1266	16.1585
0.0132	0.1686	1.0118	0.2041	0.3634	0.4221	0.1262	16.2489
0.0086	0.2798	1.5838	0.1021	0.2964	0.4411	0.2020	29.2876
0.0107	0.2802	1.5838	0.1061	0.2974	0.4417	0.2025	29.6989
0.0125	0.2800	1.5847	0.1052	0.2974	0.4418	0.2026	29.6679
0.0448	0.1137	0.8497	0.0771	0.3894	0.3639	0.1710	18.4251
0.0449	0.1141	0.8498	0.0766	0.3893	0.3634	0.1706	18.2956
0.0451	0.1140	0.8497	0.0767	0.3887	0.3628	0.1706	18.2751
0.0636	0.0793	0.7904	0.0739	0.5409	0.3246	0.2191	15.1891
0.0636	0.0793	0.7895	0.0736	0.5402	0.3238	0.2187	15.0717
0.0637	0.0793	0.7906	0.0738	0.5395	0.3235	0.2185	15.1090
0.0703	0.1374	2.2247	0.1087	0.4867	0.4520	0.1934	20.0025
0.0703	0.1376	2.2236	0.1081	0.4874	0.4523	0.1939	19.9721
0.0697	0.1376	2.2244	0.1079	0.4870	0.4518	0.1936	19.9968
0.0221	0.0858	0.5236	0.0224	0.2188	0.3019	0.1380	10.9806
0.0245	0.0897	0.5343	0.0236	0.2207	0.3049	0.1392	11.0525
0.0239	0.0870	0.5305	0.0227	0.2211	0.3052	0.1393	11.0844
0.0204	0.1522	1.9538	0.1464	0.2666	0.3649	0.1272	18.8596
0.0229	0.1525	1.0172	0.1453	0.2651	0.3628	0.1265	17.7556
0.0229	0.1524	1.0387	0.1444	0.2652	0.3628	0.1264	17.7620
0.0546	0.2556	1.2610	0.0940	0.2866	0.4231	0.2110	28.1241
0.0129	0.2558	1.2610	0.0925	0.2852	0.4223	0.2116	27.7883
0.0542	0.2555	1.2620	0.0926	0.2852	0.4224	0.2112	27.8458
0.0291	0.1283	0.6444	0.0093	0.2183	0.3328	0.1823	15.5473
0.0152	0.1284	0.6445	0.0096	0.2180	0.3322	0.1822	15.4241
0.0284	0.1282	0.6443	0.0096	0.2176	0.3315	0.1818	15.4326
0.0450	0.0748	0.5470	0.0517	0.1465	0.2087	0.1403	17.8562
0.0450	0.0748	0.5476	0.0515	0.1469	0.2093	0.1407	17.7583
0.0449	0.0749	0.5482	0.0515	0.1454	0.2080	0.1400	17.7892
0.0618	0.0704	1.6023	0.0990	0.6064	0.4492	0.2328	15.4034
0.0624	0.0706	1.6011	0.0985	0.6066	0.4490	0.2329	15.3618
0.0628	0.0708	1.6009	0.0985	0.6056	0.4483	0.2324	15.3550
0.0099	0.0295	0.3992	0.0303	0.1580	0.2586	0.0870	5.3197
0.0102	0.0008	0.3991	0.0307	0.1573	0.2573	0.0866	5.3009
0.0099	0.0006	0.3987	0.0308	0.1579	0.2583	0.0868	5.2834

Flag leaves

Varname		tech_1	tech_2	tech_3
Spitfire	S0	9.90455E-05	9.88026E-05	9.71018E-05
Spitfire	S30	7.43837E-05	7.38681E-05	7.40154E-05
Wyalkatchem	S0	9.01103E-05	9.05248E-05	9.06906E-05
Wyalkatchem	S30	6.41424E-05	6.39658E-05	6.42012E-05

Developing grains

Varname	DPA	S0_tech1	S0_tech2	S0_tech3	S30_tech1	S30_tech2
Spitfire	7	7.24205E-06	6.89078E-06	6.93469E-06	6.44705E-06	6.81042E-06
Spitfire	14	7.29908E-06	7.20837E-06	7.02695E-06	8.52393E-06	7.49416E-06
Spitfire	21	4.10227E-06	4.14252E-06	4.38398E-06	6.85779E-06	5.33324E-06
Spitfire	28	9.50135E-06	1.01802E-05	1.01425E-05	1.56897E-05	1.62693E-05
Spitfire	35	7.08976E-06	7.13173E-06	6.37633E-06	1.69322E-05	1.66226E-05
Wyalkatchem	7	4.69602E-06	4.12895E-06	3.84541E-06	3.63968E-06	3.718E-06
Wyalkatchem	14	1.0465E-05	8.14836E-06	8.49157E-06	4.28681E-06	3.40489E-06
Wyalkatchem	21	9.84106E-06	6.87503E-06	6.44517E-06	2.85529E-06	3.0839E-06
Wyalkatchem	28	6.23682E-06	6.1955E-06	6.56739E-06	1.12291E-05	8.33335E-06
Wyalkatchem	35	6.08059E-06	4.98127E-06	5.89737E-06	1.03374E-05	7.31555E-06

S30_tech3
6.62874E-06
7.45297E-06
5.62968E-06
1.57789E-05
1.56941E-05
2.77809E-06
3.48506E-06
1.21687E-06
8.02623E-06
6.18693E-06

Varname	Streat	Ntreat	glu/gli	ω_5%	ω_1,2%	α/β%	γ%	ω%	gliadins%
Livingston	S0	N25	0.2936	0.0301	0.0541	0.3727	0.3163	0.0841	0.7731
Livingston	S0	N25	0.2507	0.0237	0.0500	0.3769	0.3489	0.0738	0.7995
Livingston	S0	N25	0.1910	0.0398	0.0591	0.4054	0.3353	0.0989	0.8396
Mace	S0	N25	0.3017	0.0241	0.0435	0.3656	0.3351	0.0676	0.7682
Mace	S0	N25	0.3254	0.0266	0.0421	0.3464	0.3394	0.0687	0.7545
Mace	S0	N25	0.2355	0.0285	0.0549	0.3409	0.3851	0.0834	0.8094
Westonia	S0	N25	0.4125	0.0250	0.0478	0.3358	0.2993	0.0728	0.7080
Westonia	S0	N25	0.3712	0.0270	0.0470	0.3452	0.3101	0.0740	0.7293
Westonia	S0	N25	0.4609	0.0212	0.0478	0.3205	0.2949	0.0690	0.6845
Wyalkatchem	S0	N25	0.3349	0.0211	0.0435	0.3448	0.3398	0.0645	0.7491
Wyalkatchem	S0	N25	0.4324	0.0208	0.0401	0.3271	0.3101	0.0609	0.6981
Wyalkatchem	S0	N25	0.4118	0.0205	0.0383	0.2804	0.3692	0.0588	0.7083
Livingston	S30	N25	0.6294	0.0168	0.0404	0.2919	0.2647	0.0572	0.6137
Livingston	S30	N25	0.5967	0.0189	0.0364	0.2984	0.2725	0.0553	0.6263
Livingston	S30	N25	0.5111	0.0234	0.0424	0.3257	0.2703	0.0658	0.6618
Mace	S30	N25	0.4717	0.0182	0.0376	0.3189	0.3048	0.0559	0.6795
Mace	S30	N25	0.6137	0.0160	0.0395	0.2764	0.2879	0.0555	0.6197
Mace	S30	N25	0.9282	0.0165	0.0305	0.2015	0.2701	0.0470	0.5186
Westonia	S30	N25	0.6758	0.0129	0.0462	0.2753	0.2623	0.0591	0.5967
Westonia	S30	N25	0.9061	0.0177	0.0341	0.2279	0.2449	0.0518	0.5246
Westonia	S30	N25	0.6673	0.0157	0.0319	0.2745	0.2777	0.0476	0.5998
Wyalkatchem	S30	N25	0.6275	0.0142	0.0300	0.2852	0.2850	0.0443	0.6144
Wyalkatchem	S30	N25	0.6097	0.0203	0.0353	0.2678	0.2979	0.0556	0.6212
Wyalkatchem	S30	N25	0.9531	0.0123	0.0305	0.2213	0.2479	0.0428	0.5120
Livingston	S50	N25	0.4298	0.0055	0.0630	0.3467	0.2842	0.0685	0.6994
Livingston	S50	N25	0.4910	0.0041	0.0644	0.3464	0.2557	0.0685	0.6707
Livingston	S50	N25	0.4158	0.0050	0.0596	0.3682	0.2734	0.0647	0.7063
Mace	S50	N25	0.4187	0.0075	0.0593	0.3381	0.3000	0.0668	0.7049
Mace	S50	N25	0.4192	0.0061	0.0652	0.3203	0.3129	0.0713	0.7046
Mace	S50	N25	0.4178	0.0056	0.0623	0.3564	0.2810	0.0679	0.7053
Westonia	S50	N25	0.4056	0.0109	0.0636	0.3522	0.2848	0.0744	0.7115
Westonia	S50	N25	0.5055	0.0096	0.0535	0.3293	0.2719	0.0631	0.6642
Westonia	S50	N25	0.4885	0.0095	0.0472	0.3251	0.2900	0.0566	0.6718
Wyalkatchem	S50	N25	0.5366	0.0104	0.0399	0.3011	0.2995	0.0502	0.6508
Wyalkatchem	S50	N25	0.4620	0.0165	0.0349	0.3211	0.3115	0.0514	0.6840
Wyalkatchem	S50	N25	0.5873	0.0138	0.0386	0.2944	0.2832	0.0524	0.6300

GS1

DPA	Streat	Bio_R1	Bio_R2	Bio_R3
7	S0	1.0000	1.0000	1.0000
7	S30	0.6552	0.6071	0.3820
14	S0	0.9352	0.5664	0.5697
14	S30	0.9919	0.2379	0.7440
21	S0	0.8655	1.4557	0.9954
21	S30	1.1933	1.1134	1.0151

GS2

DPA	Streat	Bio_R1	Bio_R2	Bio_R3
7	S0	1.0000	1.0000	1.0000
7	S30	0.3622	0.7371	1.1500
14	S0	0.2567	0.2517	0.5064
14	S30	0.2137	0.1688	0.1537
21	S0	0.0846	0.2076	0.2071
21	S30	0.0328	0.1233	0.2720

Varname	Streat	Ntreat	glu/gli	ω_5%	ω_1,2%	α/β%	γ%	ω%	gliadins%
Spitfire	S0	N25	0.5442	0.0164	0.0371	0.3450	0.2491	0.0535	0.6476
Spitfire	S0	N25	0.6495	0.0115	0.0350	0.2970	0.2628	0.0464	0.6062
Spitfire	S0	N25	0.5897	0.0156	0.0344	0.3298	0.2493	0.0499	0.6290
Wyalkatchem	S0	N25	0.5623	0.0120	0.0367	0.3067	0.2847	0.0486	0.6401
Wyalkatchem	S0	N25	0.5379	0.0173	0.0291	0.2966	0.3073	0.0464	0.6502
Wyalkatchem	S0	N25	0.6442	0.0108	0.0284	0.2875	0.2815	0.0392	0.6082
Spitfire	S30	N25	0.9867	0.0103	0.0237	0.2519	0.2175	0.0340	0.5033
Spitfire	S30	N25	1.0477	0.0097	0.0282	0.2660	0.1845	0.0379	0.4883
Spitfire	S30	N25	1.1634	0.0119	0.0202	0.2288	0.2014	0.0321	0.4622
Wyalkatchem	S30	N25	0.8120	0.0086	0.0206	0.2713	0.2514	0.0291	0.5519
Wyalkatchem	S30	N25	1.3163	0.0085	0.0185	0.2113	0.1934	0.0270	0.4317
Wyalkatchem	S30	N25	1.0416	0.0086	0.0210	0.2195	0.2407	0.0296	0.4898
Spitfire	S50	N25	1.1081	0.0158	0.0301	0.2420	0.1864	0.0459	0.4744
Spitfire	S50	N25	1.2100	0.0217	0.0246	0.2156	0.1906	0.0463	0.4525
Spitfire	S50	N25	1.1533	0.0161	0.0213	0.2282	0.1988	0.0374	0.4644
Wyalkatchem	S50	N25	0.7034	0.0192	0.0238	0.2529	0.2912	0.0430	0.5870
Wyalkatchem	S50	N25	0.6710	0.0215	0.0290	0.2617	0.2862	0.0506	0.5984
Wyalkatchem	S50	N25	0.7600	0.0253	0.0284	0.2415	0.2730	0.0537	0.5682
