# Supplementary material for: Impact and mechanism of sulphur-deficiency on modern wheat farming nitrogen-related sustainability and gliadin content
Source: Commun Biol. 2021 Aug 6;4:945. doi: 10.1038/s42003-021-02458-7 (PMC8346565; doi:10.1038/s42003-021-02458-7)
Supplement: Supplementary file 14 — Reporting Summary [file 42003_2021_2458_MOESM14_ESM.pdf]

nature research

Corresponding author(s): Wujun Ma

Last updated by author(s): Jun 20, 2021

# Reporting Summary

Nature Research wishes to improve the reproducibility of the work that we publish. This form provides structure for consistency and transparency in reporting. For further information on Nature Research policies, see our Editorial Policies and the Editorial Policy Checklist.

## Statistics

For all statistical analyses, confirm that the following items are present in the figure legend, table legend, main text, or Methods section.

| n/a | Confirmed | |
|---|---|---|
| ☐ | ☒ | The exact sample size ($n$) for each experimental group/condition, given as a discrete number and unit of measurement |
| ☐ | ☒ | A statement on whether measurements were taken from distinct samples or whether the same sample was measured repeatedly |
| ☐ | ☒ | The statistical test(s) used AND whether they are one- or two-sided<br>*Only common tests should be described solely by name; describe more complex techniques in the Methods section.* |
| ☒ | ☐ | A description of all covariates tested |
| ☒ | ☐ | A description of any assumptions or corrections, such as tests of normality and adjustment for multiple comparisons |
| ☐ | ☒ | A full description of the statistical parameters including central tendency (e.g. means) or other basic estimates (e.g. regression coefficient) AND variation (e.g. standard deviation) or associated estimates of uncertainty (e.g. confidence intervals) |
| ☐ | ☒ | For null hypothesis testing, the test statistic (e.g. $F$, $t$, $r$) with confidence intervals, effect sizes, degrees of freedom and $P$ value noted<br>*Give P values as exact values whenever suitable.* |
| ☒ | ☐ | For Bayesian analysis, information on the choice of priors and Markov chain Monte Carlo settings |
| ☒ | ☐ | For hierarchical and complex designs, identification of the appropriate level for tests and full reporting of outcomes |
| ☒ | ☐ | Estimates of effect sizes (e.g. Cohen's $d$, Pearson's $r$), indicating how they were calculated |

*Our web collection on statistics for biologists contains articles on many of the points above.*

## Software and code

Policy information about availability of computer code

| | |
|---|---|
| Data collection | N/A |
| Data analysis | N/A |

For manuscripts utilizing custom algorithms or software that are central to the research but not yet described in published literature, software must be made available to editors and reviewers. We strongly encourage code deposition in a community repository (e.g. GitHub). See the Nature Research guidelines for submitting code & software for further information.

## Data

Policy information about availability of data

All manuscripts must include a data availability statement. This statement should provide the following information, where applicable:

- Accession codes, unique identifiers, or web links for publicly available datasets
- A list of figures that have associated raw data
- A description of any restrictions on data availability

The RNA-seq datasets generated during the current study are available in the NCBI SRA repository. The accession code for deposited data is PRJNA719174. The source data for all figure items is presented in Supplementary Data 11. All other datasets generated during the current study are available from the corresponding author on reasonable request.

# Field-specific reporting

Please select the one below that is the best fit for your research. If you are not sure, read the appropriate sections before making your selection.

☐ Life sciences ☐ Behavioural & social sciences ☒ Ecological, evolutionary & environmental sciences

For a reference copy of the document with all sections, see nature.com/documents/nr-reporting-summary-flat.pdf

# Ecological, evolutionary & environmental sciences study design

All studies must disclose on these points even when the disclosure is negative.

| | |
|---|---|
| Study description | Using large-scale field and glasshouse experiments and transcriptomic and metabolomic analyses to explore the impacts of sulphur fertilization on sulphur-deficient wheat crop and its gluten composition |
| Research sample | Five cultivars: The wheat cultivars used in the current study, including two cultivars in 2014 and 2015 glasshouse experiments and four cultivars in 2014 field trial, are highly adopted Australian bread wheat cultivars that are widely cultivated in Australia. Flag leaves and developing grains from these cultivars are used. |
| Sampling strategy | All plots and pots respectively from 2014 field trial and 2014 glasshouse experiment were harvested at grain maturity. The peduncle parts from Spitfire and Wyalkatchem in three sulphur treatments from three biologically independent replicates in 2014 glasshouse experiment were collected for measurement. The flag leaves of Spitfire in S0 and S30 treatments from three biologically independent replicates in 2015 glasshouse experiment were collected at the stage of flowering for measuring glutamine synthetase (GS) activity. The developing grains from the middle row of the main tiller head of Spitfire at each developing stage in S0 and S30 treatments from three biologically independent replicates in 2015 glasshouse experiment were taken for RNA-seq assay, GS activity assay, and free amino acid assay.<br>For the 2014 field trial, a total of 36 mature grain samples were obtained and analysed from 36 plots for agronomic trait and gluten component, including 4 cultivars (Livingston, Mace, Westonia and Wyalkatchem), 3 sulphur treatments (S0, S30 and S50) and 3 replicate plots for each sulphur treatment. For the 2014 glasshouse experiment, the peduncle sample size and the mature grain sample size for agronomic trait and gluten component are both 18, including 2 cultivars (Spitfire and Wyalkatchem), 3 sulphur treatments (S0, S30 and S50) and 3 replicate pots for each sulphur treatment. The flag leaf samples and developing grain samples collected from 2015 glasshouse experiment were used for mechanism studies, including RNA-seq assay, GS activity assay and free amino acid assay. For RNA-seq assay, grain samples of Spitfire were collected from 3 developing grain stages (7, 14 and 21 DPA) of 2 sulphur treatments (S0 and S30) with 3 biologically independent replicates (or 3 pots), making it a total of 18 samples. For GS activity and free amino acid assay, a total of 6 flag leaf samples of each cultivar (Spitfire or Wyalkatchem) were collected from 2 sulphur treatments (S0 and S30) with 3 biologically independent replicates (or 3 pots) for each sulphur treatment to measure GS activity in flag leaves; a total of 30 developing grain samples of each cultivar (Spitfire or Wyalkatchem) from 5 grain developing stages (7, 14, 21, 28 and 35 DPA) of 2 sulphur treatments (S0 and S30) with 3 biologically independent replicates (or 3 pots) were used for conveying GS activity dynamics in developing grain; a total of 36 developing grain samples of Spitfire were collected from 6 grain developing stages (7, 14, 21, 28, 35 and 42 DPA) of 2 sulphur treatments (S0 and S30) with 3 biologically independent replicates (or 3 pots) to extract free amino acid from developing grain. The flag leaf samples for measuring GS activity as well as the developing grain samples for conveying GS activity dynamics and extracting free amino acid were subject to the commonly used sample subpooling strategy, making it 2 pooled flag leaf samples of each cultivar for measuring GS activity in flag leaves, 10 pooled developing grain samples of each cultivar for conveying GS activity dynamics in developing grains, and 12 pooled developing grain samples of Spitfire for extracting free amino acid from developing grain, followed by 3 technical repeats for each. |
| Data collection | The grain yield and protein yield of the sample from 2014 field trial were measured after harvesting and threshing, collected by the staff in Katanning research station of Department of Primary Industries and Regional Development.<br>The grain yield and peduncle traits of the sample from 2014 glasshouse experiment were measured after harvesting, collected by the author Zitong Yu.<br>The nitrogen-use efficiency for the sample from 2014 field trial and 2014 glasshouse experiment was determined by the grain yield or protein yield produced by per kg of nitrogen applied, calculated as the grain yield or protein yield divided by the amount of N applied at 25 kg/ha. |
| Timing and spatial scale | The sample from 2014 field trial was collected when the grain was mature.<br>For the sample from 2014 and 2015 glasshouse experiments, each pot was watered every morning with demineralized water. The flag leaf from 2015 glasshouse experiment was collected at flowering time, and the developing grains from the middle row of the main tiller head of cultivar Spitfire in 2015 glasshouse experiment were collected at 7-day intervals, with sample collection starting at 7 days post-anthesis (DPA) and ending at 42 DPA, including 7, 14, 21, 28, 35, and 42 DPA. |
| Data exclusions | No data exclusions. |
| Reproducibility | Three biologically independent replicates were used in this study. |
| Randomization | The pots in 2014 and 2015 glasshouse experiments were placed based on randomized complete block design. The sample from each pot was collected randomly. |
| Blinding | The samples from 2014 and 2015 glasshouse experiments were collected randomly. |

Did the study involve field work? ☒ Yes ☐ No

## Field work, collection and transport

| | |
|---|---|
| Field conditions | The soil sulphur of the trial site was measured at 4.0 mg/kg. |
| Location | The field trial was conducted in the main Australian wheat production zone, the Western Australia Wheat-Belt region Katanning. |
| Access & import/export | The field trial site can be accessed without restrictions. No import/export issues. |
| Disturbance | No. |

# Reporting for specific materials, systems and methods

We require information from authors about some types of materials, experimental systems and methods used in many studies. Here, indicate whether each material, system or method listed is relevant to your study. If you are not sure if a list item applies to your research, read the appropriate section before selecting a response.

### Materials & experimental systems

| n/a | Involved in the study |
|---|---|
| ☒ | ☐ Antibodies |
| ☒ | ☐ Eukaryotic cell lines |
| ☒ | ☐ Palaeontology and archaeology |
| ☒ | ☐ Animals and other organisms |
| ☒ | ☐ Human research participants |
| ☒ | ☐ Clinical data |
| ☒ | ☐ Dual use research of concern |

### Methods

| n/a | Involved in the study |
|---|---|
| ☒ | ☐ ChIP-seq |
| ☒ | ☐ Flow cytometry |
| ☒ | ☐ MRI-based neuroimaging |
